# *BrPP5.2* Overexpression Confers Heat Shock Tolerance in Transgenic *Brassica rapa* through Inherent Chaperone Activity, Induced Glucosinolate Biosynthesis, and Differential Regulation of Abiotic Stress Response Genes

**DOI:** 10.3390/ijms22126437

**Published:** 2021-06-16

**Authors:** Muthusamy Muthusamy, Jong Hee Kim, Suk Hee Kim, So Young Park, Soo In Lee

**Affiliations:** 1 Department of Agricultural Biotechnology, National Institute of Agricultural Sciences (NAS), Rural Development Administration, Jeonju 54874, Korea; biotech.muthu@gmail.com (M.M.); gllmon@naver.com (J.H.K.); hbhb0@naver.com (S.H.K.); psy0203@korea.kr (S.Y.P.); 2 Division of Horticultural Biotechnology, Hankyung National University, Anseong 17579, Korea

**Keywords:** thermotolerance, chaperone activity, heat stress response genes, glucosinolate, protein phosphatase 5, *Brassica rapa*

## Abstract

Plant phosphoprotein phosphatases are ubiquitous and multifarious enzymes that respond to developmental requirements and stress signals through reversible dephosphorylation of target proteins. In this study, we investigated the hitherto unknown functions of *Brassica rapa* protein phosphatase 5.2 (*BrPP5.2*) by transgenic overexpression of *B. rapa* lines. The overexpression of *BrPP5.2* in transgenic lines conferred heat shock tolerance in 65–89% of the young transgenic seedlings exposed to 46 °C for 25 min. The examination of purified recombinant BrPP5.2 at different molar ratios efficiently prevented the thermal aggregation of malate dehydrogenase at 42 °C, thus suggesting that BrPP5.2 has inherent chaperone activities. The transcriptomic dynamics of transgenic lines, as determined using RNA-seq, revealed that 997 and 1206 (FDR < 0.05, logFC ≥ 2) genes were up- and down-regulated, as compared to non-transgenic controls. Statistical enrichment analyses revealed abiotic stress response genes, including heat stress response (HSR), showed reduced expression in transgenic lines under optimal growth conditions. However, most of the HSR DEGs were upregulated under high temperature stress (37 °C/1 h) conditions. In addition, the glucosinolate biosynthesis gene expression and total glucosinolate content increased in the transgenic lines. These findings provide a new avenue related to BrPP5.2 downstream genes and their crucial metabolic and heat stress responses in plants.

## 1. Introduction

Plants adapt to developmental requirements, depending on stress and other internal and external signals, by switching on and off critical regulatory proteins/enzymes. The phosphorylation and dephosphorylation of target proteins are among the primary critical regulatory mechanisms found in plants. Both phosphorylation and dephosphorylation are reversible post-translational modifications controlled by kinases and phosphatases, respectively [1]. These enzymes are equally crucial in regulating almost all pathways, including signal transduction, stress responses, and cellular control circuitry [2]. Plant phosphatases can dephosphorylate serine/threonine (Ser/Thr phosphatases), tyrosine (tyrosine phosphatases), or both residues (dual-specificity phosphatases) of the side chains of the target protein. Ser/Thr phosphatases consist of Ser/Thr-specific protein phosphatases (phosphoprotein phosphatases (PPP)), metal ion-dependent protein phosphatases (PPM), and aspartate-dependent phosphatases [3]. PPPs are ubiquitous [4] and multifarious enzymes that include PP1, PP2 (PP2A), PP3 (PP2B), PP4, PP5, PP6, and PP7 enzymes [5,6]. Of these, PP1 and PP2A are the most prevalent, accounting for more than 85% of the total cellular phosphatase activity in plants [1,7]. The other subclasses (PP4, PP5, PP6, and PP7) have been studied less in plants owing to their relatively low phosphatase activity. Nonetheless, studies focusing on the physiological roles of some those enzymes have shown their prominent association with several signal transduction pathways, stress responses/tolerance, plant growth, and development [6,8,9,10,11]. 

A recent investigation of PP4 shed light on its association with miRNA biogenesis [12]. PP5s are crucial for disease resistance, light detection [10], thermotolerance [11,13], tetrapyrrole-mediated plastid signaling [14], hormone production, and flowering [9,10]. Similarly, PP6 is essential for flowering time modulation [9], auxin efflux [15], ABA signaling, and seed germination [7,16]. In addition, PP7s have been shown to participate in the blue light signaling [17], thermotolerance [18], and phytochrome signaling [19] pathways. Many more roles of PPPs can be discovered if their complex substrate interaction/multiple targets are known. The role of phosphatase inhibitors is crucial for identifying their biological significance [1,8]. Among PPPs, PP2A, PP1, and PP5 are sensitive to okadaic acid, cantharidin, and microcystin [8,11,20]. Of the PPPs, PP5 is structurally unique. It has a regulatory domain (tetratricopeptide repeat (TPR)) and a catalytic domain (a phosphatase) at the N and C terminals of the same polypeptide [21]. The TPR domain and the C-terminal helix αJ jointly maintain free PP5 in an autoinhibited conformation [11]. The interaction between the TPR domain with Hsp90 and fatty acids, such as arachidonic acid, induces PP5 activity [22], while interaction with okadaic acid and microcystin inhibits [4]. Similarly, TPR interactions with various receptor protein complexes determine the plant responses, depending on the perceived signals. As more frequent episodes of climatic anomalies occur globally, it is essential to identify potential candidate genes that confer stress tolerance in plants. *Brassica rapa PP5* could also participate in abiotic stress tolerance, but this is yet to be studied.

Our in silico study with the *B. rapa* genome identified two *PP5* genes, which were likely the result of genome duplication events. In this study, we attempted to characterize *BrPP5.2* (Bra004718) in heat shock stress responses, using transgenic technology. A comparative analysis of the heat tolerance efficacy of *BrPP5.2* overexpressing transgenic (BrPP5.2OX) lines showed that their relative thermotolerance was significantly higher than that of the existing heat-tolerant, sensitive, and non-transgenic controls. In addition, we found that recombinant BrPP5.2 possesses holdase chaperone-like activities, thus preventing the thermal aggregation of a given substrate, MDH, suggesting that BrPP5.2-inherent chaperone activities could help impart thermotolerance in transgenic plants. Additionally, several hundreds of BrPP5.2 downstream genes and their differential expression in transgenic and non-transgenic control lines were profiled using Illumina-based RNA-seq technology. We also found that *BrPP5.2* overexpression induced glucosinolate (GSL) biosynthesis-related gene expression in transgenic lines. Quantitative HPLC analyses of GSLs in transgenic lines confirmed that GSL concentration was higher in these transgenic lines than that in controls under normal and high temperature stress conditions. This study revealed that *BrPP5.2* overexpression confers thermotolerance in *B. rapa* through multiple mechanisms.

## 2. Results

### 2.1. BrPP5.2 Expression Profiling in Different Tissues, Hormones, Stress Agents and Development of BrPP5.2-Overexpressing Transgenic Lines

**Figure 1:**
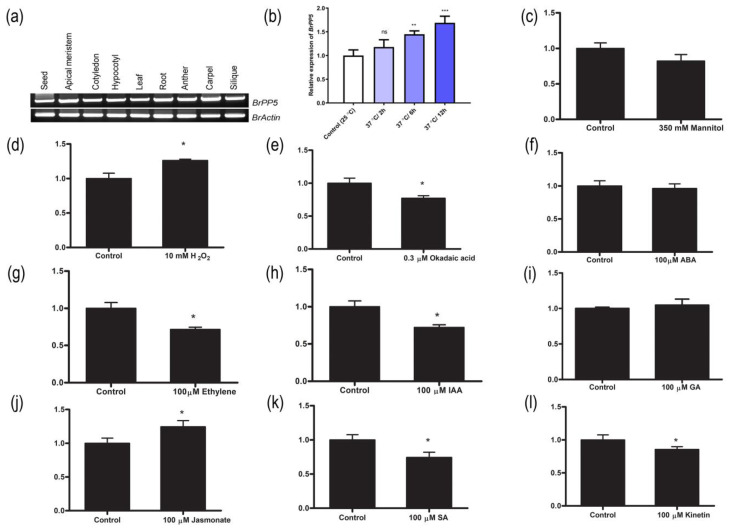
*BrPP5.2* expression profiling across different tissues and treatment. (**a**) RT-PCR results showed the expression of *BrPP5.2* in vegetative (apical meristems, cotyledon, hypocotyl, leaf, and root) and reproductive (seed, anther, carpel, and silique) tissues of *B. rapa*. (**b**–**l**) The quantitative RT-PCR results showing the relative expression pattern of *BrPP5.2* in *B. rapa* exposed to a high temperature (37 °C) at different times (**b**), exogenously supplied 350 mM Mannitol (**c**), 10 mM hydrogen peroxide (**d**), AtPP5 inhibitor, okadaic acid (0.3 µM) (**e**), and phytohormones (100 µM each) such as abscisic acid (**f**)**,** ethephon (**g**), indole acetic acid (**h**), gibberellins (**i**), methyl jasmonate (**j**), salicylic acid (**k**) and kinetin (**l**). Each column represents the normalized gene expression values derived from qRT-PCR replicates, while error bars denote the standard deviations. One-way ANOVA (Dunnett’s multiple comparisons) test was performed in GraphPad Prism 8.0.2 to determine the significant variation from controls grown under optimal growth conditions. Ns = non-significant; * = *p* < 0.05; ** = *p* < 0.01; *** = *p* < 0.001. *BrACT2* was used for gene expression normalization.

We found two genes (*Bra004718, Bra016856*) encoding characteristic protein phosphatase 5 (*BrPP5.2*) in the *B. rapa* genome in this study. The *Bra004718*-encoded *BrPP5.2* sequence shared higher similarity (88.32%) with *AtPP5* (*AT2G42810*) than *Bra016856* (88.34% with 77% query coverage). In addition, a high nucleotide similarity of 89.98% was observed between Bra004718 and Bra016856. To decipher the role of *BrPP5.2* in stress responses and its role in plant growth and development in *B. rapa*, we investigated the Bra004718-encoded *BrPP5.2* in the present study. The deduced protein sequence, comprising 484 amino acids, has a characteristic N-terminal tetratricopeptide repeat (TPR) domain (positioned between 13 and 114 AA residues) containing three TPR motifs and a C-terminal phosphatase domain (162–467 AA). Sequence homology analysis showed that the AA of BrPP5.2 shares 93.39% identity with the protein phosphatase 5.2 of *A. thaliana*. The subcellular localization prediction (http://localizer.csiro.au/ accessed on 12 November 2020) showed that it could be targeted at the nucleus. This protein’s theoretical pI and molecular weight (https://web.expasy.org/protparam/ accessed on 12 November 2020) were calculated to be around 5.67 and 54.80 kDa, respectively. The tissue-specific expression analysis of *BrPP5.2* showed its obvious presence in the apical meristem, cotyledon, hypocotyl, leaf, root, pollen, carpel, silique, and seeds (Figure 1a).

**Figure 2:**
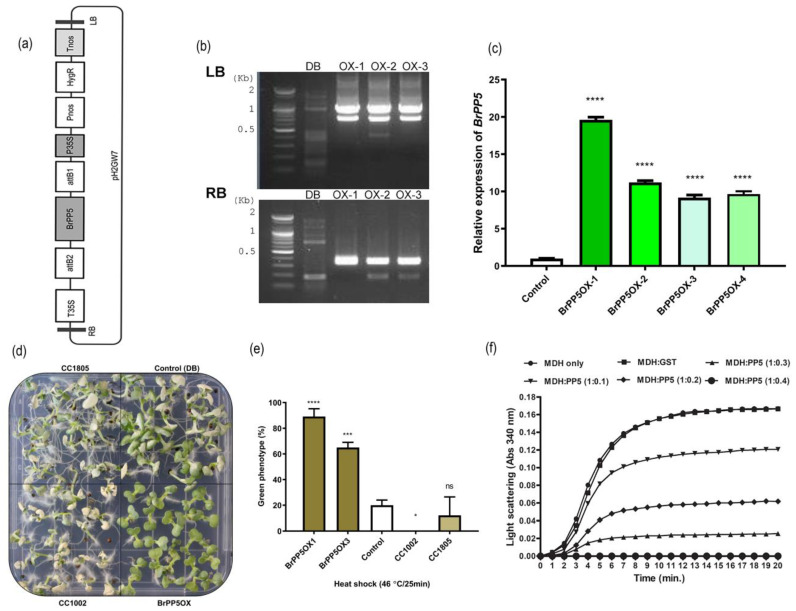
Development of *BrPP5.2* overexpressing transgenic *B. rapa* lines and the phenotyping studies. (**a**) The simplified genetic expression cassette used for the overexpression of *BrPP5.2* by Agrobacterium-mediated genetic transformation. (**b**) The results of T-DNA flanking sequencing of the selected transgenic lines confirming the transgenicity and identifying the transgene integration site in the host genome. (**c**) The transgene overexpression confirmation with non-transgenic control at the transcript level by a relative quantification method using qRT-PCR assay. *BrACT2* was used for gene expression normalization. (**d**,**e**) The heat shock stress (at 46 °C for 25 min) phenotyping of in vitro grown *BrPP5.2* overexpressing transgenic lines (BrPP5OX) along with non-transgenic controls (DB) and heat-tolerant (CC1805) and heat-sensitive (CC1002) *B. rapa* cultivars (**d**)**,** while (**e**) represents the heat stress tolerance efficiencies of the respective test cultivars. (**f**) The graph illustrates the holdase type chaperone efficiencies of recombinant BrPP5.2 (provided at different molar ratios) in preventing heat stress (42 °C) induced aggregation of a substrate, malate dehydrogenase (MDH), for 1–20 min. Ns = non-significant; * = *p* < 0.05; *** = *p* < 0.001; **** = *p* < 0.0001.

qRT-PCR analysis showed that the exogenous application of phytohormones (Figure 1f–i), abiotic stress signals (Figure 1b–d), and PP5 inhibitors is responsible for the *BrPP5.2* expression changes (Figure 1e). Exposure to heat stress (37 °C) for 6–12 h significantly induced the expression of *BrPP5.2*. Moreover, the exogenous supply of methyl jasmonate and H_2_O_2_ increased the expression of *BrPP5.2* in young *B. rapa* seedlings. Under similar conditions, IAA, salicylic acid, ethylene, and kinetin significantly reduced *BrPP5.2* expression when compared to that in control plants, whereas ABA and gibberellic acid either did not alter *BrPP5.2* expression or caused statistically negligible changes. Exposure to okadaic acid also reduced *BrPP5.2* expression significantly. The protein-coding region (1.455 kb) was genetically overexpressed in *B. rapa* plants using Agrobacterium-mediated genetic transformation to understand the biological significance of *BrPP5.2* (Figure 2a–f). qRT-PCR-based expression profiling showed that the transgene expression in the selected transgenic lines was 9.64- to 19.60-fold higher than that in the control lines (Figure 2c). Furthermore, the T-DNA flanking sequencing with the selected lines confirmed that transgene integration occurred near Bra019016 of ChrA06 (the distance between the integration site and 3’ downstream is approximately 0.284 kb) (Figure 2b). The function of Bra019016 is yet to be elucidated. The phenotypic differences between transgenic (T3) and control lines during heat stress and in the presence of known PP5 inhibitors (okadaic acids) were studied, and the possible changes in the molecular mechanisms of transgenic lines were investigated in this study.

### 2.2. BrPP5.2 Overexpression Results in Enhanced Heat Shock Stress Tolerance in Transgenic Lines

The heat stress responses of BrPP5.2OX seedlings, non-transgenic controls (DB), and heat stress-tolerant (CC1805) and heat stress-sensitive (CC1002) *B. rapa* cultivars grown in MSA plates were investigated by exposing them to 46 °C for 25 min. Phenotyping analyses revealed that BrPP5.2OX showed better survivability by retaining the green phenotypes of 65–89% of the total population, and this percentage was significantly higher than that observed in the case of the controls, which was 20% (Figure 2d,e). The survival rates of CC1805 and CC1002 cultivars were 12% and 0%, respectively. The results indicate that *BrPP5.2* overexpression confers heat shock stress tolerance in transgenic lines.

The growth parameters of transgenic lines in MSA medium containing 350 mM mannitol and okadaic acid were investigated. The addition of 0.3 μM okadaic acid (PP5 inhibitor) inhibited root growth in mannitol MSA medium, indicating that the suppression of PP5 likely impaired osmotic tolerance in transgenic lines (Figure S1).

### 2.3. BrPP5.2 Possesses Holdase Chaperone Activity

The ability of BrPP5.2, which functions as a holdase chaperone, was examined using MDH as a substrate (Figure 2f). The ability of BrPP5.2 to prevent the thermal aggregation of MDH at a higher temperature for a given time is considered a performance indicator for holdase chaperones. The thermal aggregation of MDH decreased with increasing concentration of BrPP5.2 proteins, and aggregation was prevented completely at a 1:0.4 molar ratio of MDH to BrPP5.2. The holdase chaperone activity was not detected for GST, while the holdase chaperone activity of BrPP5.2 was detected at all molar ratios, and it was higher than that of the control protein, GST. This study confirms that BrPP5.2 possesses holdase chaperone activity, which is likely helpful in the acquisition of thermotolerance by the plants.

### 2.4. Transcriptomic Signatures of BrPP5.2OX Lines

**Figure 3:**
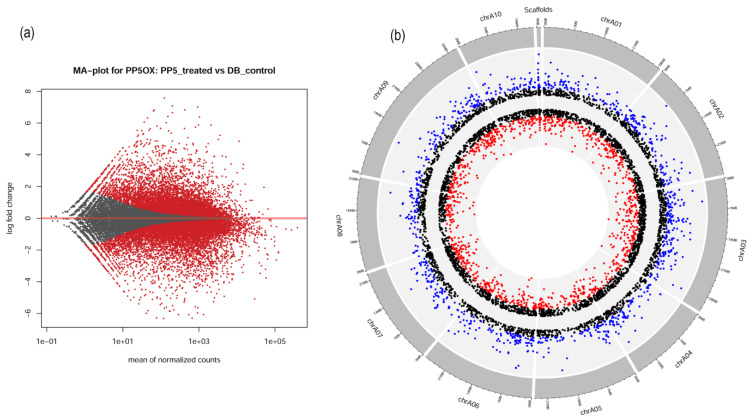
The transcriptomic signatures of *BrPP5.2* overexpressing transgenic *B. rapa* lines. (**a**) The MA plot represents the differentially expressed genes (DEGs) (FDR < 0.05) derived from RNA sequencing data of transgenic lines compared to those of non-transgenic controls. (**b**) The circus plot depicts DEGs across the 10 *B. rapa* chromosomes. The color codes represent the significance levels of DEGs. The red spots indicate the downregulated DEGs (logFC ≤ |−2|; FDR < 0.05), and blue spots (logFC ≥ |2|; FDR < 0.05) represent the upregulated DEGs in BrPP5.2OX lines, while black spots represent the non-significant DEGs observed in differential gene expression analysis between transgenic and control lines.

To understand the basis of *BrPP5.2-*mediated thermotolerance, the transcriptomic dynamics of transgenic lines were measured and compared with those of the controls. Additionally, the transcriptome signatures will help to infer the comprehensive lists of *BrPP5.2* downstream genes. A total of 78.04 million clean/filtered reads from BrPP5.2OX libraries and 88.54 million clean/filtered reads from the control libraries were produced, mapped onto the *B. rapa* genome sequence, and assembled into 40,689 non-redundant unigenes (Data S1). Differential gene expression analyses using edgeR and DESeq2 revealed that the expression of 5803 transcriptional units was significantly altered (logFC ≥ 1; *p* < 0.05) in the transgenic seedlings (Figure 3a; Data S1 and S2). The raw sequence reads were made available in the NCBI Sequence Read Archive (SRA) under BioProject accession number PRJNA701981. (https://dataview.ncbi.nlm.nih.gov/object/PRJNA701981?reviewer=kok65dlecmc6vqot8m29enqght accessed on 12 November 2021).

### 2.5. Chromosome-Wise Distribution of DEGs

The common DEGs (5969) with logFC > |1| and FDR < 0.05 were plotted using their genomic coordinates in 10 chromosomes using a Circos plot to infer the gene expression changes at the chromosome level (Figure 3b; Data S3 and S4). To distinguish the degree of fold change, three different color codes (red = genes with logFC < |−2|, blue = logFC > |2|, and black = logFC between |−2| and |2|) were used. The DEGs were distributed among all 10 chromosomes and in the scaffolds (128 DEGs). Chr3 was found to have the highest number of DEGs (880), followed by chr9 (855 DEGs) and chr6 (636). The lowest number of DEGs was found in chr10 (405 DEGs). Most downregulated DEGs were observed with chr9, while chr3 had the highest number of upregulated genes. Incidentally, chr9 was the longest chromosome found in *B. rapa*, while chr4 was the shortest (Figure 3b).

### 2.6. BrPP5.2 Overexpression in Transgenic Lines Causes Changes in Metabolic and Abiotic Stress Responses

**Figure 4:**
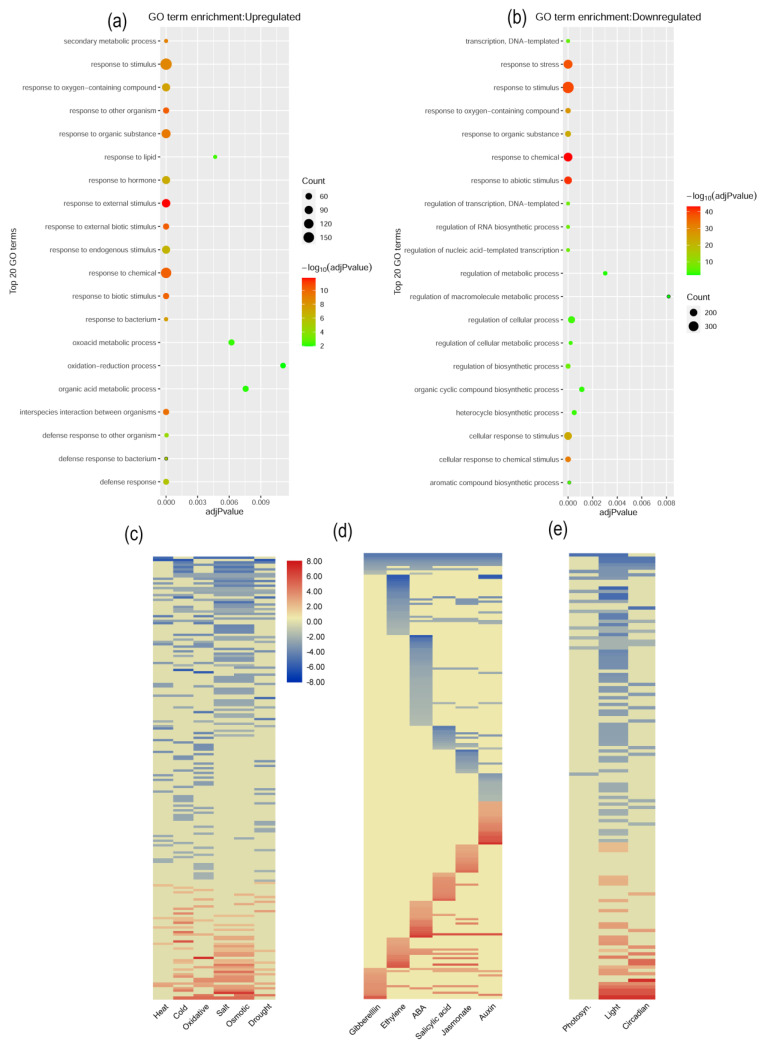
Functional enrichment and classification of differentially expressed genes of thermo-tolerant BrPP5.2OX transgenic lines. (**a**,**b**) Statistical enrichment analysis of corresponding gene ontologies of significantly (logFC ≥ 2; FDR < 0.05) upregulated (**a**) (652 DEGs) and (**b**) downregulated (864 DEGs) genes reported in thermotolerant transgenic lines by the gProfiler tool. (**c**–**e**) Heat maps represent the expression pattern of predominant DEGs responsible for abiotic stress responses (**c**) (heat, cold, oxidative, salt, osmotic, and drought stress), hormonal responses (**d**) (gibberellins, ethylene, salicylic acid, jasmonate, and auxin), (**e**) photosynthesis, light stimulus response, and circadian genes of transgenic lines.

The statistical enrichment analysis of 1516 DEGs (logFC ≥ 2; FDR < 0.05) with known GO terms revealed that transcripts associated with the molecular biology components, multiple biological processes, and cellular components were enriched (Figure 4a–e). In particular, DEGs related to abiotic stimulus responses (stress, temperature stimulus, osmotic stress, cold, water deprivation, blue light, and cellular stress response), hormones (auxin and salicylic acid), biotic stimuli, circadian rhythm, response to lipids, the GSL metabolic process under the biological processes category, the DNA-binding transcription factor activity of the molecular functional category, and the cell wall in the cellular component categories were overrepresented in transcriptomic changes between the transgenic and control lines. Among the known KEGG pathways, the photosynthesis–antenna protein-related, MAPK signaling pathway-related, and secondary metabolite biosynthesis-related were predominant. However, according to the WikiPathways database, GSL biosynthesis is enriched in transgenic lines.

To infer the expression pattern of *BrPP5.2* downstream genes and dissect their positive or negative functions in transgenic lines, upregulated (652) and downregulated (864) DEGs were ordered according to their expression levels and separately analyzed using the gProfiler tool (Figure 4a; Data S5). The statistical enrichment of upregulated DEGs indicated that genes involved in biological processes such as plant responses to external stimuli, chemicals, biotic stimuli, and hormones (jasmonic acid and salicylic acid) were elevated in transgenic lines. Additionally, genes governing molecular fractions such as the secondary metabolic process, circadian rhythm, chitinase activity and cellular components like secretory vesicles, and cell walls were enriched. In terms of pathway analysis, GSL biosynthesis is likely to be induced in BrPP5.2OX lines, as indicated by the upregulated DEGs. A similar study with downregulated DEGs revealed that abiotic stimuli/stress response (heat, temperature, cold, and oxidative stress), nitrogen compound response, cellular stress response, and ethylene response processes of transgenic plants were negatively regulated (Figure 4b; Data S5). Among the molecular components category, genes critical for regulating DNA-binding transcription factor activity, transcription regulatory regions, and photosystem I were negatively regulated in BrPP5.2OX lines.

### 2.7. Stress-, Hormone-Response, and Development-Related Gene Expression Patterns

The functional enrichment of upregulated DEGs (652) showed that DEGs responsive to the stimuli (GO: 0050896) (175), those responsive to chemicals (GO: 0042221) (144), and those responsive to hormones (GO: 0009725) (86) were some of the predominant examples of the enrichment of DEGs in BrPP5.2OX lines (Data S5). A similar analysis of downregulated DEGs (864) showed that DEGs responsive to biological processes such as stimuli (GO:0050896) (384), stress (GO:0006950) (255), and abiotic stimuli (GO:0009628) (206) were highly reduced under normal conditions in BrPP5.2OX lines. The enrichment of DEGs responsive to stress (GO:0006950) (255), cellular stress (GO:0033554) (106), temperature (GO: GO:0009266) (67), osmotic stress (GO: 0006970) (48), oxidative stress (GO: 0006979) (47), salt stress (GO: GO:0009651) (41), water deprivation (GO: GO:0009414) (32), auxins (GO:0009733) (33), and cold stress (GO: GO:0009409) (42) was also found to be reduced under normal conditions (Data S5). In contrast, DEGs responsive to external biotic stimuli (GO:0043207) (54), defense (GO:0006952) (51), jasmonic acid (GO:0009753) (25), salicylic acid (GO:0009751) (17), GSL metabolic processes (GO:0019760) (19), gibberellins (GO:0009739) (14), and photoperiodism (GO:0009648) (10) were induced in transgenic *B. rapa*.

### 2.8. Expression Pattern of Phytohormone-Responsive DEGs

A total of 205 hormone-response genes have been reported. Of these, 77, 54, 51, 46, 37, and 24 DEGs are known to respond to ABA, jasmonate, ethylene, SA, auxins, and gibberellins, respectively (Figure 4d; Data S6). Expression pattern analysis showed that many of the genes responsive to ABA (55 DEGs; 71.4%) were downregulated in heat shock-tolerant BrPP5.2OX lines under normal conditions. It is not clear whether the downregulation of ABA response-related genes could facilitate thermotolerance. However, a recent study showed that ABA negatively modulates heat tolerance in rice varieties by regulating energy homeostasis [23]. Similarly, most of the DEGs responsive to ethylene (68.6%), auxins (56.4%), jasmonic acid (53.7%), and SA were downregulated. However, 58.33% of DEGs responsive to gibberellins were upregulated. In addition, there was a reduction in the expression of type-A Arabidopsis response regulator (A-ARR), mitogen-activated protein kinase 6 (*MPK6*), touch 4 (*TCH4*), and JASMONATE-ZIM-DOMAIN PROTEIN (*JAZ*) classes. In contrast, auxin/indole -3-acetic acid family (*AUX/IAA*), small auxin upregulated RNA (*SAUR*), PYRABACTIN RESISTANCE/PYRABACTIN RESISTANCE-LIKE (*PYR/PYL*), protein phosphatase type 2C (*PP2C*), ethylene response (*ETR*), and pathogenesis-related protein 1 (*PR1*) class genes were induced.

### 2.9. Expression Pattern of Photosynthesis-, Light-, and Circadian-Related Genes

The present study reported 85 DEGs responsive to light stimulus, 33 responsive to circadian rhythm, and 10 responsive to photosynthesis and light harvesting in photosystem I (Figure 4e; Data S6). Notably, 100% of the DEGs responsive to photosynthesis/light harvesting in photosystem I, 72.9% of those responsive to light stimulus, and 57.5% of those responsive to circadian rhythm showed reduced expression under optimal growth conditions, indicating negative regulation by *BrPP5.2*. In particular, DEGs (Bra002999, Bra000708, Bra025297, Bra005424, Bra033022, Bra008216, Bra027083, Bra030182, Bra010807, and Bra026099) that participate in light harvesting in photosystem I showed reduced expression. Moreover, phytochrome-mediated photoresponse DEGs (PHYTOCHROME- INTERACTING FACTOR7 (*PIF7*; Bra012972), EARLY-PHYTOCHROME-RESPONSIVE1 (*EPR1*; Bra025914)) showed reduced expression.

### 2.10. Expression Profiling of Temperature Stimulus Response (TSR) DEGs at High Temperature Stress Conditions

In total, 89 TSR DEGs were identified (Figure 4c; Data S6). Of these, 35 DEGs were known to be responsive to heat stress, whereas 62 DEGs were known to be responsive to cold stress. Eight common DEGs were responsive to the heat and cold stress pathways. Approximately 83% of the DEGs responsive to heat stress (29 of 35; 83%) were significantly downregulated compared to the observed non-transgenic control lines. Under similar conditions, the expression of 41 of 62 (66%) DEGs responsive to cold stress was also reduced. Six DEGs, the 26.5 kDa class *p*-related heat shock protein gene (*HSP26.5-P; Bra020865*), pleiotropic drug resistance 12 (*PDR12; Bra026124*), touch 4 (*TCH4; Bra010292*), cytochrome P450 71B2 (*CYP71B2; Bra026937*), tudor domain-containing protein (*Bra029309*), and ABA insensitive 1 (*ABI1; Bra010441*) from the heat stress pathway, were upregulated in BrPP5.2OX lines.

**Figure 5:**
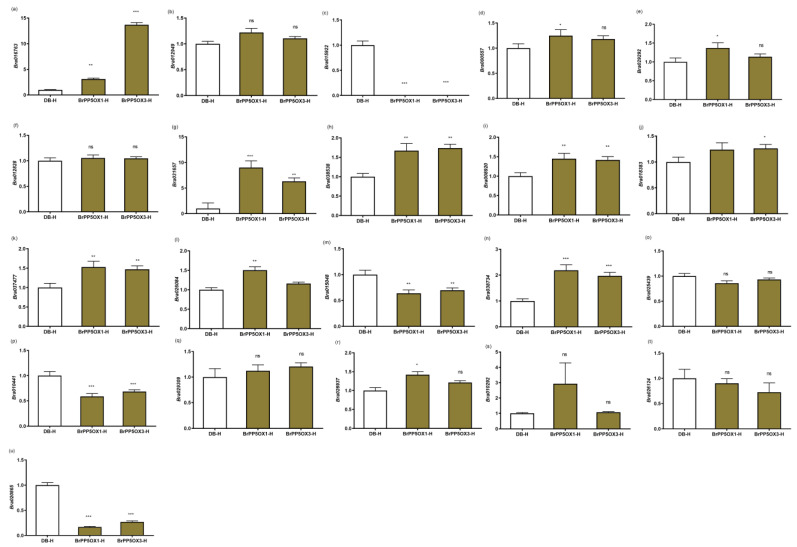
Expression profiling of temperature stimulus response genes under heat stress in transgenic and control lines. (**a**–**u**) The column charts represent the relative expression pattern of *DDF1* (Bra016763) (**a**), *HSP17.4-CIII* (Bra012949) (**b**), *ATHSP101* (Bra015922) (**c**), *ATHSFA2* (Bra000557) (**d**), *AT-HSFB2A* (Bra029292) (**e**), *AT-HSFA7A* (Bra012828) (**f**), *BIP3* (Bra031657) (**g**), *MIPS2* (Bra038538) (**h**), *HSP17.6II* (Bra008920) (**i**), *HSP17.6B-CI* (Bra018383) (**j**), *ROF2* (Bra037477) (**k**), *Hsp70b* (Bra026084) (**l**), *MBF1C* (Bra015048) (**m**), *HSP70* (Bra038734) (**n**), *TCH2* (Bra025439) (**o**), *ABI1* (Bra010441) (**p**), *Tudor domain-containing protein*, *AT5G61780* (Bra029309) (**q**), *CYP71B2* (Bra026937) (**r**), *TCH4* (Bra010292) (**s**), *DR12* (Bra026124) (**t**), and *HSP26.5-P* (Bra020865) (**u**) measured by quantitative RT-PCR assay in cDNA libraries derived from 2-month-old transgenic and control lines (*n* = 3 or more) exposed to 37 °C for 1 h. Each column represents the normalized gene expression values derived from qRT-PCR replicates, while error bars denote the standard deviations. One-way ANOVA (Dunnett’s multiple comparisons) test was performed in GraphPad Prism 8.0.2 to determine the significant variation from controls grown under optimal growth conditions. Ns = non-significant; * = *p* < 0.05; ** = *p* < 0.01; *** = *p* < 0.001. *BrACT2* was used for gene expression normalization.

However, under high temperature (37 °C/1 h) stress conditions, most of the expression patterns of TSR genes were upregulated (Figure 5a–u). A total of 22 DEGs were investigated for their expression patterns in BrPP5.2OX lines and controls under high temperature stress conditions using qPCR. Of these, 11 DEGs comprising DWARF AND DELAYED FLOWERING 1 (DDF1; Bra016763), *ATHSFA2* (Bra000557), *AT-HSFB2A* (Bra029292), *BIP3* (Bra031657), MYO-INOSITOL-1-PHOSPHATE SYNTHASE 2 (*MIPS2*; Bra038538), 17.6 KDA CLASS II HEAT SHOCK PROTEIN (*HSP17.6II*; Bra008920), 17.6 kDa class I small heat shock protein (*HSP17.6B-CI*; Bra018383), *ROF2* (Bra037477), *Hsp70b* (Bra026084), *HSP70* (Bra038734), and CYTOCHROME P450 71B2 (*CYP71B2*; Bra026937) were significantly upregulated in transgenic lines. Conversely, four DEGs, *ATHSP101* (Bra015922), MULTIPROTEIN BRIDGING FACTOR 1C (*MBF1C*; Bra015048), ABA INSENSITIVE 1 (*ABI1*; Bra010441), and *HSP26.5-P* (Bra020865), were significantly downregulated. The expression patterns of other genes, such as *HSP17.4-CIII* (Bra012949), *AT-HSFA7A* (Bra012828), *TCH2* (Bra025439), tudor domain-containing protein, *AT5G61780* (Bra029309), *TCH4* (Bra010292), and *PDR12* (Bra026124), were not significantly altered. Surprisingly, five of the six upregulated DEGs found under optimal conditions were downregulated under high temperature stress conditions (Figure 4c and Figure 5a–u; Data S6).

### 2.11. Functional Classification of Significant DEGs

To decipher the regulatory functions of BrPP5.2, all significant (logFC ≥ 2; FDR < 0.05) DEGs (2203) were classified into several gene families based on the popular BRAD database [24,25]. This study identified 314 transcription factors (TFs), 37 auxin genes, 26 GSL genes, 12 resistance genes, 5 anthocyanin genes, and 6 flower genes (Data S7). Among the TFs, 198, which contributed 63% of the total TFs, were downregulated in BrPP5.2OX lines (Figure S2). TF profiles mainly comprised the AP2-EREBP, MYB-related, MYB, WRKY, and NAC families. Of these, the expression of a significant portion of AP2-EREBP (76%), MYB-related (81%), MYB (60.7%), and NAC TFs (56.5%) significantly reduced. However, the expression of TF families such as WRKY (54%), C2H2 (63%), and C2C2-CO-like (71%) was upregulated in comparison with that in non-transgenic controls. A significant portion of the auxin (75.6%) and GSL (80.7%) genes were upregulated in transgenic plants under normal conditions. Among the resistance genes, 9 of the 12 DEGs were downregulated. Similarly, three and two anthocyanin and flower DEGs were downregulated.

### 2.12. Changes in the Expression Pattern of GSL Biosynthesis-Related Genes and GSL Content in Transgenic Lines

**Figure 6:**
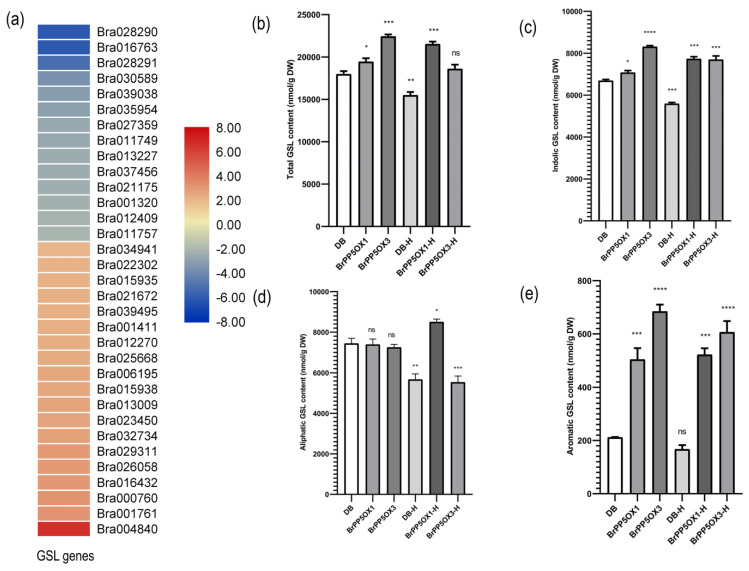
Expression pattern of GSL biosynthesis-related genes and GSL concentration in transgenic and control lines under normal and heat stress conditions. (**a**) The heatmap depicts the expression pattern of GSL genes in BrPP5 overexpressing transgenic lines compared to non-transgenic controls. (**b**–**e**) The graphs represent the UHPLC results (described in the methods section), revealing glucosinolate types and their concentration (nmol/g DW) in aerial tissues of 2-month-old transgenic and control lines (*n* = 15 or more) grown under optimal and high temperature/heat (37 °C for 1 h) stress conditions. (**b**) Total GSL content in non-transgenic controls (DB), transgenic lines (BrPP5OX1, BrPP5OX3), heat-stressed non-transgenic controls (DB-H), and heat-stressed transgenic lines (BrPP5OX1-H, BrPP5OX3-H). (**c**) Aliphatic GSL content in non-transgenic controls (DB), transgenic lines (BrPP5OX1, BrPP5OX3), heat-stressed non-transgenic controls (DB-H), and heat-stressed transgenic lines (BrPP5OX1-H, BrPP5OX3-H). (**d**) Indolic GSL content in non-transgenic controls (DB), transgenic lines (BrPP5OX1, BrPP5OX3), heat-stressed non-transgenic controls (DB-H), and heat-stressed transgenic lines (BrPP5OX1-H, BrPP5OX3-H). (**e**) The aromatic GSL content in non-transgenic controls (DB), transgenic lines (BrPP5OX1, BrPP5OX3), heat-stressed non-transgenic controls (DB-H), and heat-stressed transgenic lines (BrPP5OX1-H, BrPP5OX3-H). Each column represents the mean values derived from UHPLC duplicates, while error bars denote the standard deviations. One-way ANOVA (Dunnett’s multiple comparisons test) was performed using GraphPad Prism 8.0.2 to determine the significant variation from non-transgenic controls grown under optimal growth conditions. Ns = non-significant; * = *p* < 0.05; ** = *p* < 0.01; *** = *p* < 0.001; **** = *p* < 0.0001.

According to the BRAD database, at least 102 *B. rapa* genes are known to participate in GSL metabolism. We report 26 GSL genes with differential expression patterns in the BrPP5.2OX lines (Figure 6a; Data S7; Figure S3). Among them, 21 DEGs comprising *CYP83A1* (Bra032734), *CYP83B1* (Bra034941), *CYP79F1* (Bra026058), *AOP2* (Bra000848; Bra018521), *MAM1* (Bra029355), *MAM3* (Bra013009), *ST5a* (Bra015935), *ST5b* (Bra015938; Bra027880), *ST5c* (Bra025668), *BCAT-4* (Bra022448; Bra001761), *BAT5* (Bra029434; Bra000760), *UGT74C1* (Bra005641), *MYB28* (Bra012961; Bra029311), *MYB122* (Bra008131), *GSTF9* (Bra022815), and *IPMDH1* (Bra023450) were upregulated. *ST5b* (Bra027880) followed by *MYB122* (Bra008131) had the highest expressions of 5.06- and 4.45-fold over the controls. Five other DEGs representing *GSL-OH* (Bra021670)*, CHY1* (Bra018392)*, GSL-OH* (Bra021671), *MYB34* (Bra035954), and *ST5b* (Bra027623) were downregulated.

We also used quantitative HPLC methods to investigate the effect of the differential regulation of GSL genes on GSL content in transgenic lines (Figure 6b–e; Data S8 and S9). The results revealed that the total GSL, indolic GSL, and aromatic GSL contents were significantly (*p* < 0.05) higher in transgenic lines than in non-transgenic controls. In contrast, no significant changes in aliphatic GSL content were observed between the control and transgenic lines. Altogether, seven aliphatic (prostaglandin (PGT), glucoraphanin (GRA), glucoalyssin (GAS), gluconapoleiferin (GNA), gluconapin (GNP), glucobrassicanapin (GBN), and glucoerucin (GER)), four indolic (glucobrassicin (GBS), 4-hydroxyglucobrassicin (4-HGB), 4-methoxyglucobrassicin (4-MTGB), and neoglucobrassicin (NGB)), and one aromatic (gluconasturtiin (GNT)) GSLs were detected. Of these, GBS, 4-MTGB, NGB, and GNT levels were elevated in the transgenic lines (Data S8). A similar analysis with high temperature stressed lines showed that total, indolic, and aromatic GSLs remained higher in transgenic lines than in the stressed non-transgenic controls (Figure 6b–e; Data S8).

### 2.13. Differential Splicing of Potential Targets

In addition to the changes in the expression of downstream genes, the overexpression of *BrPP5.2* also induced changes in the splicing pattern of 669 genes (FDR < 0.05), either directly or indirectly, in transgenic lines, compared to the controls (Data S10). Transducin family protein (Bra021603.1), CONSTANS-like protein-related (Bra030669.1), elongation factor 1-alpha/EF-1-alpha (Bra030707.1), *NEDD1* (neural precursor cell expressed, developmentally down-regulated gene 1; Bra034848.1), *PP5.2* (protein phosphatase 5.2; Bra004718.1), *RPL18AA* (60s ribosomal protein L18A-1; Bra027325.1), *YSL3* (yellow stripe-like 3; Bra003047.1), unknown protein (Bra037364.1), *TIFY10B* (Bra015880.1), and *PROT1* (proline transporter 1; Bra000154.1) are some of the top-ranked genes which were differentially spliced. In addition, 53 of 669 differentially spliced genes were found with significant variations in expression. Of these, 21 genes were upregulated, while 32 were downregulated.

### 2.14. Brassica, Transgenic Specific DEGs

We found 247 functionally unknown/novel DEGs, would-be species- or genus-specific genes with significant variations in transcript expression (Data S11). Of these, 69 DEGs were found with expression changes of two or more folds compared to non-transgenic controls. The magnitude of the expression level ranged between |+| 9.23-|−|7.03-fold. These may be involved in key biological, molecular, and cellular processes mediated by *BrPP5.2* in *B. rapa*. This study also identified 76 DEGs that were unique to the transgenic lines. Their expression changes varied between |+|4.59- and |+|10.97-fold. Functional annotation with Arabidopsis gene models identified the general functions of 55 DEGs, while 21 DEGs were functionally unknown. GO terms-based functional enrichment (69 DEGs) showed that the pectin catabolic process (BP), UDP-glucosyltransferase activity, quercetin 7-O-glucosyltransferase activity (MF), and pollen tube, plasma membrane-bounded cell projection (CC) were enriched in transgenic lines.

## 3. Discussion

*B. rapa* is an economically important vegetable crop, and its sustainable production is often threatened by heat stress, as frequent episodes of climate anomalies continue to occur amidst increasing global warming. Thus, it is essential to identify critical regulators of abiotic stress for the development of stress-resilient crops. Previous studies have shown that protein phosphatases play a crucial role in stress signaling pathways [26]. Plant phosphatases have been explored in earlier studies owing to their crucial roles in plant stress responses and other critical cellular activities; however, the same is not true for PP5 and a few other plant phosphatases as they exhibit low activity in plants [4]. In this study, we attempted to characterize one of the BrPP5.2 encoding genes originating from the *ChrA05:1504445-1507252* region in the *B. rapa* genome. Additionally, we examined the full spectrum of BrPP5.2 downstream genes and their expression patterns in transgenic lines. BrPP5.2 has characteristic and unique structural features responsible for its interaction with targets/proteins when required by plants [4,11,21,22]. In particular, TPR, consisting of a 34-amino acid repeat domain in BrPP5.2, possesses intrinsic chaperone activity that possibly enables it to selectively bind with targets. BrPP5.2, which is highly homologous to *AtPP5.2,* was significantly suppressed by okadaic acids. The potential interaction of BrPP5.2 with targets possibly altered the transcriptomic signatures of several hundreds of genes. Nonetheless, ten existing interaction partners of BrPP5.2, predicted by the STRING database, do not show transcript expression changes.

Heat stress responses are a complex quantitative trait involving multiple regulatory players that are responsible for transient or permanent modifications in order to cope with adverse conditions depending on the stress intensity and duration. Endogenous phytohormones also play an essential role in regulating a diverse array of potential stress-responsive genes. In this study, we investigated the hormonal modulation of ubiquitous *BrPP5.2* by exogenous phytohormone applications and found that IAA, salicylic acid, and ethylene effectively modulated *BrPP5.2* expression. Among abiotic stress conditions, high-temperature stress, hydrogen peroxide/oxidative stress, and mannitol-induced drought stress significantly enhanced the expression of *BrPP5.2*. The hormonal and abiotic stress responses of *BrPP5.2* indicate their potential role in abiotic stress responses.

### BrPP5.2 Overexpression Confers Thermotolerance in Transgenic Lines Through Multiple Regulatory Mechanisms

Agrobacterium-mediated genetic transformation for the overexpression of *BrPP5.2* yielded stable transgenic lines. T-DNA flanking sequencing verified the transgenicity and identified the transgene integration site in the transgenic lines. Meanwhile, qPCR-based expression analysis confirmed the *BrPP5.2* overexpression pattern in transgenic lines when compared with that in the controls. qPCR revealed that *BrPP5.2* was overexpressed by 9- to 19-fold in the selected transgenic plants, which is ideal for its functional characterization. The promising thermotolerance of BrPP5.2OX homozygous T3 lines confirms that *BrPP5*.2 overexpression results in thermotolerance. The thermotolerance efficiency was higher than that of the known heat stress-tolerant *B. rapa* cultivars. To understand the basis of *BrPP5*.2-mediated thermotolerance, we examined the potential role of recombinant BrPP5.2 as a chaperone, previously demonstrated with phosphatases in other crops [13]. The different molar ratios of the BrPP5.2 recombinant protein produced by *E. coli* efficiently prevented the aggregation of the substrate, MDH, during exposure to high temperatures, thus confirming its role as a chaperone during heat stress. Therefore, we conclude that the holdase-like chaperone activity of BrPP5.2 contributes to thermotolerance in transgenic lines.

Nonetheless, prior to this study, precise information about the BrPP5.2 downstream genes and their possible role in plant stress responses, growth, and development was not available. To gain this knowledge, we measured the transcriptomic dynamics of BrPP5.2OX lines and compared them with those of non-transgenic controls using RNA sequencing. Altogether, the results showed that *BrPP5.2* overexpression altered (logFC ≥ 2, FDR < 0.05) the expression pattern of 5.41% of the total protein-coding genes reported in this study. Of these, 1206 DEGs, constituting 54.74% of total DEGs, showed significantly reduced expression, indicating that *BrPP5*.2 is a dominant-negative regulator of downstream genes. The DEGs were distributed across all chromosomes, thus indicating that *BrPP5.2* overexpression induced broad, genome-wide expression changes in transgenic plants. Cross-species annotation with Arabidopsis identified the gene ontologies of 1516 DEGs mainly enriched with abiotic stimulus response genes, auxin and SA-responsive (hormones) genes, biotic stimulus response genes, and GSL metabolic process-related genes. The enriched transcripts imply that *BrPP5*.2 is crucial in governing the molecular basis of plant abiotic stress responses according to endogenous and exogenous signals.

Further careful classification and ranking of these biological processes based on the expression patterns of the DEGs and their significance levels indicate that the overexpression of *BrPP5.2* reduced the expression of abiotic stress response genes, including that of HSR genes, under normal conditions. This result is indicative of the role of BrPP5.2 in the negative regulation of heat stress response genes. These HSR response gene groups consisted of several classes of HSPs (*HSP21*, *HSP17.6C-CI*, *DNAJ*, *ATHSP23.6-MITO*, *HSP17.6II*, *HSP17.6B-CI*, *HSP17.6II*, *HSP17.6B-CI*, and *HSP17.4-CIII*), HSFs, and other genes with heat stress-protective roles in plant cells. The effect of the reduced expression of these genes in plant development under optimal growth conditions merits further research.

Nonetheless, the expression patterns of HSR genes during high temperature stress and their association with BrPP5.2-mediated thermotolerance are not clear. Hence, 24 genes identified from large-scale transcriptomic studies were analyzed for their expression patterns under high temperature stress (37 °C/1 h) conditions by qPCR assay. Heat stress upregulated most HSR genes at the transcriptional level, which usually had opposite expression patterns under standard growth conditions. The overexpression of many of these HSR genes, such as DDF1 [27], *ATHSFA2* [28], *AT-HSFB2A* [29], *BIP3* [30], *MIPS2* [31], *HSP17.6II* [32], *HSP17.6B-CI* [33], *Hsp70b* [34], *HSP70* [35], and CYP71B2 [36], would facilitate improved tolerance to heat stress. Thus, we conclude that the overexpression of HSR genes during heat stress may contribute to BrPP5.2-mediated heat stress tolerance. The precise mechanism by which heat stress modulates BrPP5.2 downstream targets needs to be investigated, although temperature-dependent dephosphorylation of targets can control large-scale transcriptomic changes in plants [37].

In addition, we found that 21 of 26 GSL biosynthesis-associated genes were induced at the transcript level in transgenic lines. This induced expression pattern of GSL genes was also consistent with the total GSL content in transgenic lines, which implies that BrPP5.2 can regulate GSL content by modulating the GSL biosynthesis-related genes through yet-to-be-identified mechanisms. Of the upregulated DEGs, the upregulation of *MYB28* [38]; *CYP83B1*, *MYB122*, and *ST5a* [39]; *ST5b* and *ST5c* [40]; *MAM3* [41]; and *MAM1* and *CYP83A1* [42] was shown to increase the GSL content. Other genes such as *CYP79F1*, *BCAT4*, *AOP2, UGT74C1, BAT5* [43], and sulfotransferases (*ST5b*, *ST5c*) [44] have been shown to participate in various steps of GSL biosynthesis and its modification. Studies have also shown that Brassicas with a high GSL content show better heat stress tolerance [45]. Therefore, we expect that the increased GSL accumulation observed during high temperature stress can aid plants in acquiring thermotolerance. Although the mechanism by which GSL contributes to thermotolerance is yet to be identified, it is clear that *BrPP5.2* participates in GSL biosynthesis.

A recent study that dealt with heat stress phenotypes of lentil cultivars revealed that cytochrome P450s, known for their ROS scavenging roles, are induced in tolerant cultivars [46]. Although no previous *B. rapa* cytochrome P450 implications are available, five cytochromes P450 (Bra026937, Bra039495, Bra034941, Bra032734, and Bra026058) were induced in the thermotolerant BrPP5.2OX lines, which may indicate that the positive correlation exists between cytochrome P450 and thermotolerance in this study. Plant phosphatases have been shown to work antagonistically to protein kinases [26]. This relationship was partially true for *BrPP5.2* and protein kinases, as was evident in the transgenic lines wherein 49% of the total protein kinases showed significantly reduced expression.

Other fractions of the transcriptome signatures of the transgenic lines revealed that several other molecular elements governing light responses, photosynthesis, and circadian rhythms were negatively regulated. In particular, the DEGs participating in light harvesting at photosystem I showed reduced expression at the transcriptional level. In addition, 57–72% of the total DEGs associated with light stimulus response and circadian rhythm regulation showed significantly reduced expression in transgenic lines. Hence, it is presumed that *BrPP5.2* overexpression may lead to reduced energy metabolism. In another study, overexpression of the PP5 isoform, PAPP5, was positively correlated with greater photoresponsiveness and enhanced expression of light-inducible genes [4,10]. In contrast, *BrPP5*.2 overexpression resulted in a 73% reduction in the expression of total light stimulus response genes.

We also identified several DEGs specific to Brassica and BrPP5.2OX lines that are also likely to play a role in thermotolerance. The functional characterization of these candidate genes will add to our understanding of BrPP5.2-mediated thermotolerance. Nonetheless, BrPP5.2-mediated thermotolerance is possible through inherent heat chaperone activities of BrPP5.2, enhanced GSL content, and the modulation of abiotic stress response genes.

## 4. Materials and Methods

### 4.1. Plant Materials and Stress Treatments

*B. rapa* (‘DH03’) seedlings were used in this study. Eight-day-old seedlings (*n* = 5) grown in a hydroponic system were supplemented with phytohormones (each at 100 μM concentration) such as abscisic acid (ABA), indole-3-acetic acid (IAA), ethylene (Ethephon), kinetin, gibberellic acid (GA3), salicylic acid (SA), and methyl jasmonate (JA) in a growth chamber. Concurrently, plant PP5 inhibitors, i.e., okadaic acid (0.3 μM), hydrogen peroxide (10 mM), and D-mannitol (350 mM), were also added, and the seedlings were incubated for three consecutive days. Similarly, high- and low-temperature stresses were imposed by incubating seedlings in growth chambers at 37 °C for 6 h and 4 °C for 6 h, respectively. Seedlings grown in MS solution were used as controls. All the seedlings from the treatments were harvested, frozen in liquid nitrogen, and stored at 80 °C before molecular analysis. 

### 4.2. Expression Profiling of BrPP5.2 during Exogenous Application of Phytohormones and Stress Agents and Tissue-Specific Expression

Total RNA (2 μg) was extracted from each sample using RNeasy Plant Mini Kits (Qiagen, Germany), and cDNA was prepared in 20 μL reactions with amfiRivert cDNA Synthesis Platinum Master mix according to the manufacturer’s protocol (GenDEPOT, Baker, TX, USA). For *BrPP5.2* gene expression profiling, qRT-PCR was performed using the CFX96^TM^ Real-Time PCR Detection System (Bio-Rad, Hercules, CA, USA) with a set of *BrPP5.2-*specific primers (Table S1) along with AccuPower^®^2X GreenStar Master Mix (Bioneer, Daejeon, Korea). The qPCR conditions were as follows: 95 °C for 5 min, followed by 40 cycles of 95 °C for 15 s and 56 °C for 30 s. *BrActin2* was used as an internal control. For tissue-specific expression analysis, semi-qRT-PCR was performed using the cDNAs derived from tissue samples, including apical meristem, cotyledon, hypocotyl, leaf, and root, pollen, carpel, silique, and seeds.

### 4.3. Designing of the BrPP5.2 Overexpression Construct and Development of Transgenic B. rapa Lines

The full-length coding region of *BrPP5.2* (1.455 kb) was amplified by the attB1 site attached to a specific primer set (Table S1) from the cDNA library of the DH line of *B. rapa* and inserted into the pH2GW7 vector using the Gateway™ Cloning system (Invitrogen, Waltham, MA, USA). The construct was transformed into *B. rapa* lines using *Agrobacterium tumefaciens* (strain GV3101) to generate transgenic plants with *BrPP5.2* overexpression (BrPP5.2OX). As mentioned previously, transgene integration sites in the host genome were identified using the T-DNA flanking sequencing method [47].

### 4.4. Phenotyping of BrPP5.2OX Lines for Heat Shock Stress Tolerance

The seeds of two selective BrPP5.2OX lines and heat-tolerant, heat-sensitive, and non-transgenic control lines were surface-sterilized and plated on half-strength Murashige and Skoog Agar (MSA) medium in triplicate (*n* = 20 or more). After 1-day-stratification at 4 °C under dark conditions, the seeds containing MSA plates were incubated in a controlled plant tissue culture room (16 h light at 25 °C and 8 h dark at 23 °C; light intensity: 100 to 120 μmol m^−2^ s^−1^) for germination. After 9-day post germination, the seedlings and plates were wrapped in a laboratory film, submerged in a water bath at 46 °C for 25 min, and then returned to optimal growth conditions for recovery. After two days of recovery, the survival rate of each line across replicates was measured by counting the green phenotypes.

### 4.5. Expression and Purification of Recombinant BrPP5.2 and Analysis of Holdase Chaperone Activity

*BrPP5.2* was amplified by PCR (F-gaattcCATGGAGACTA AGAATGAGAACTCTG; R- ctcgagTTAGTTGAACATCCTCATA AAATTGCTTG) from a *B. rapa* cDNA library and cloned into the pGEX-4T-3 vector (GE Healthcare, Piscataway, NJ, USA). The resultant construct was verified by nucleotide sequencing and then transformed into the *E. coli* strain BL21-CodonPlus (Agilent Technologies, Santa Clara, CA, USA). Glutathione-Sepharose 4B (Peptron, Daejeon, Korea) was used to purify the GST-tagged fusion BrPP5.2 protein expressed in pGEX-4T-3 by following the protocols and eluted by thrombin cutting (Thrombin Cleavage Kit, BioVision, Milpitas, CA, USA). Recombinant protein expression was induced by incubating the cultures (OD_600_ = 0.5) with 0.4 mM isopropyl-ß-D-thiogalactoside (IPTG) for 3 h at 30 °C and 200 rpm. The protein concentration was determined according to the Bradford method using BSA as a standard. The protein quality was assessed by loading and running the recombinant BrPP5 on 12% SDS-PAGE after protein extraction. As previously described [13], holdase chaperone activity was measured by combining MDH with various concentrations of BrPP5.2 in 50 mM HEPES-KOH (pH 8.0) buffer. The thermal aggregation of MDH in the presence of BrPP5.2 was monitored by measuring turbidity at 340 nm using a DU800 spectrophotometer (Beckman, Brea, CA, USA).

### 4.6. Genome-Wide Analysis of Expression Changes of BrPP5.2 Downstream Genes by RNA-Seq

Total RNA was extracted from whole seedlings (100 mg powdered tissues) of *BrPP5.2*-overexpressing transgenic lines and control lines (*n* = 20) using RNeasy Plant Mini Kit (Qiagen, Germany) according to the manufacturer’s instructions. To perform RNA quality control, RNA was quantified spectrophotometrically using a NanoDrop 2000 spectrophotometer (Thermo Scientific Co., Waltham, MA, USA), and RNA integrity (RIN) was assessed using a Bioanalyzer 2100 (Agilent Technologies, Santa Clara, CA, USA). Five micrograms of each RNA sample was used to generate six cDNA libraries (TrueSeq Stranded mRNA Prep Kit) containing inserts that were approximately 150–200 bp in size. For RNA sequencing, 101-nucleotide paired-end sequencing (*n* = 3) was conducted using an Illumina NovaSeq6000 platform (Illumina, San Diego, CA, USA) at C&K Genomics (C&K genomics Inc., Seoul, Korea). The raw RNA reads (~4.6GB or more for each sample) were filtered and trimmed using FastQC [48] and the Trimmomatic v0.39 Toolkit [49] to remove low-quality bases (>30) and adapter sequences. The preprocessed reads were aligned to *B. rapa* v.1.0 and annotation gene model v.1.0, both downloaded from https://www.plants.ensembl.org/Brassica_rapa/info/index/ (accessed on 12 November 2020), using HISAT2 (v2.1.0) [50] and default parameters. Transcriptome quantification was performed using FeatureCount (v2.0.1) [51] to calculate the transcript read counts. Raw read counts were normalized, and differential gene expression analysis was performed using edgeR (v4.0.1) [52].

We also used the DESeq2 (galaxy v2.11.40.6) package to conduct differential expression analysis [53]. Common DEGs derived from both tools with FDR < 0.05 and fold change (logFC) ≥ |2| were considered significant and were used for further analyses. Additionally, differential splicing was detected based on differences in the log-fold changes between exons for the same gene. Alternative splicing analysis was performed on each gene using the diffSpliceDGE [54] function from the edgeR package (v4.0.1). Genes with FDR < 0.05 were considered to be differentially spliced genes.

### 4.7. Gene Ontology, Functional Annotation, Statistical Enrichment, and Pathway Analyses

Identification of gene ontology (GO) terms, functional annotation, and functional enrichment of DEGs were achieved using DAVID (v6.8) [55,56] or gProfiler [57] tools after the conversion of the *B. rapa* gene IDs into Arabidopsis (TAIR) gene IDs. To perform functional enrichment with gProfiler, first, the gene IDs of the selected DEGs were submitted to the g: Convert tool (https://biit.cs.ut.ee/gprofiler/convert accessed on 12 November 2020), and their equivalent TAIR IDs, with their corresponding FDR values, were fed into g:Gost at https://biit.cs.ut.ee/gprofiler/gost (Version. e101_eg48_p14_baf17f0; accessed on 16 December 2020, 4:52:47 PM). The g:SCS threshold was chosen with a user threshold of 0.05 for the analysis. Data sources of GO, Kyoto Encyclopedia of Genes and Genomes (KEGG), and WikiPathways were utilized for this purpose. In total, 1516 DEGs (logFC ≥ 2; FDR < 0.05) with known GO terms were assigned as unordered input, while the upregulated (652) and downregulated (864) DEGs with respective FDR values were separately fed as ordered queries for this purpose. The enriched pathways identified in the BrPP5.2OX lines were selectively visualized along with the gene expression patterns and the corresponding color codes as user input with the reference pathway in the KEGG database (https://kegg.jp/kegg-binshow_pathway?map00966 accessed on 12 November 2020).

### 4.8. qRT-PCR-Based Relative Quantification of Temperature-Response DEGs in Heat Stress

To understand the expression pattern of the temperature response (21 DEGs) during heat stress, qRT-PCR was employed. Around 2-month-old transgenic and control lines grown under greenhouse conditions were transferred to a growth chamber and exposed to 37 °C for 1 h. After the treatment, the aerial parts were collected and used for total RNA extraction using the RNeasy Plant Mini Kit (Qiagen, Germany). Five micrograms of total RNAs from each line was reverse-transcribed into cDNA in 20 μL reaction mixtures using the amfiRivert cDNA Synthesis Platinum Master Mix according to the manufacturer’s protocol (GenDEPOT). The 1:20 diluted cDNAs were used as a template in gene expression studies using the CFX96^TM^ Real-Time PCR Detection System (Bio-Rad). Primer sequences and annealing conditions are provided in Table S1. *BrActin2* (FP-CTCAGTCCAAAAGAGGTATTCT; RP-GTAGAATGTGTGATGCCAGATC) was used as an internal control for normalization of gene expression.

### 4.9. Analysis of GSL Content in Transgenic and Control Seedlings under Optimal and High Temperature Stress Conditions

GSL extraction and ultra-high-performance liquid chromatography (UHPLC) analyses were performed as previously described [58]. The freeze-dried powder (2 g) samples derived from control and heat-stressed wild and transgenic seedlings (as described in this paper) were used for GSL investigation (Methods S1). The GSL profile identification and quantification were carried out by comparing the retention times, response factors, and peak areas of the desulfated GLS standard mixtures. The individual GSL concentration is expressed as nmol g^−1^ dry weight.

## 5. Patents

BrPP5 overexpression transgenic *B. rapa* lines for its thermotolerance efficacies were filed for Korean patent (Application No.10-2019-0158736).

## Data Availability

This manuscript includes the necessary data either as figures or as supplementary data. The raw sequence reads were made available in the NCBI Sequence Read Archive (SRA) under BioProject accession number PRJNA701981. (https://dataview.ncbi.nlm.nih.gov/object/PRJNA701981?reviewer=kok65dlecmc6vqot8m29enqght accessed on 12 November 2020).

## Supplementary Materials

The following are available online at https://www.mdpi.com/article/10.3390/ijms22126437/s1, Figure S1: The root phenotypes of BrPP5OX lines in the presence of 350 mM Mannitol with 0.3 µM of okadaic acid and without okadaic acid. Figure S2: The expression profiles of TFs in transgenic lines. Figure S3: The reference glucosinolate biosynthesis pathway mapped (indicated with blue color) with differentially expressed genes of *B. rapa*. Table S1: The list of primers used for qRT-PCR-based expression profiling of temperature stimulus response genes, Methods S1: The glucosinolate extraction methods of *Brassica rapa*. Data S1: List of differentially expressed genes identified in BrPP5OX transgenic B. rapa using edgeR tool. Data S2: List of differentially expressed genes identified in BrPP5OX transgenic B. rapa using DEseq2 tool. Data S3: List of differentially expressed genes commonly observed in edgeR and DEseq2 tools. Data S4: Chromosomal distribution of significant DEGs. Data S5: GO terms-based functional enrichment of up- and down-regulated DEGs. Data S6: List of genes known to be involved in abiotic stress, hormone response and energy metabolism derived from differential gene expression data. Data S7: Classification of DEGs according to BRAD database. Data S8: UPLC-based quantification results of individual glucosinolate content in transgenic and wild type lines under optimal and high-temperature stressed conditions. Data S9: The representative chromatogram of standard glucosinolate mixtures and chromatograms of each line obtained from UPLC analysis of glucosinolate measurement in *Brassica rapa*. Data S10: List of differentially spliced genes identified in BrPP5OX transgenic B. rapa lines in comparison with wild type lines. Data S11: List of Brassica- and transgenic-specific DEGs, expression pattern, and some of their functional enrichment data.
