# Supplementary material for: BrPP5.2 Overexpression Confers Heat Shock Tolerance in Transgenic Brassica rapa through Inherent Chaperone Activity, Induced Glucosinolate Biosynthesis, and Differential Regulation of Abiotic Stress Response Genes
Source: Int J Mol Sci. 2021 Jun 16;22(12):6437. doi: 10.3390/ijms22126437 (PMC8234546; doi:10.3390/ijms22126437)
Supplement: Supplementary file 1 [file ijms-22-06437-s001.zip › Captions-Supplemental.pdf]

Supplemental Figure S1: The root phenotypes of BrPP5OX lines in the presence of 350mM Mannitol with 0.3μM of okadaic acid and without okadaic acid

Supplemental Table: The list of primers used for qRT-PCR based expression profiling of temperature-stimulus response genes

Supplemental Data: List of differentially expressed genes identified in BrPP5OX transgenic B. rapa lines in comparison with wild type lines. This list includes several key features of differential gene expression data, including the gene IDs, their annotation, expression in logFC, p-values, FDR values derived from edgeR and DEseq2 tools

Supplemental Data: The UPLC based quantification results of individual glucosinolate content in transgenic and wild type lines under optima and high-temperature stressed conditions.

Supplemental Data: The representative chromatogram of standard glucosinolate mixtures and chromatograms of each lines obtained from UPLC analysis of Glucosinolate measurement in *Brassica rapa*.

Supplemental Data: The reference Glucosinolate Biosynthesis pathway mapped (indicated with blue color) with differentially expressed genes of *B. rapa*.

Supplemental Data: List of genes known to be involved in abiotic stress, hormone response and energy metabolism derived from differential gene expression data.

Supplemental Data: The chromosomal distribution of significant DEGs

Supplemental Data: The GO terms based functional enrichment of key DEGs with up- and down-regulation by gProfiler tool.

Supplemental Data: The list of brassica- and transgenic-specific DEGs, expression pattern, and some of their possible roles in Brassica rapa.
