# Supplementary material for: BrPP5.2 Overexpression Confers Heat Shock Tolerance in Transgenic Brassica rapa through Inherent Chaperone Activity, Induced Glucosinolate Biosynthesis, and Differential Regulation of Abiotic Stress Response Genes
Source: Int J Mol Sci. 2021 Jun 16;22(12):6437. doi: 10.3390/ijms22126437 (PMC8234546; doi:10.3390/ijms22126437)
Supplement: Supplementary file 1 [file ijms-22-06437-s001.zip › Chromatogram.pptx]

## Slide 1
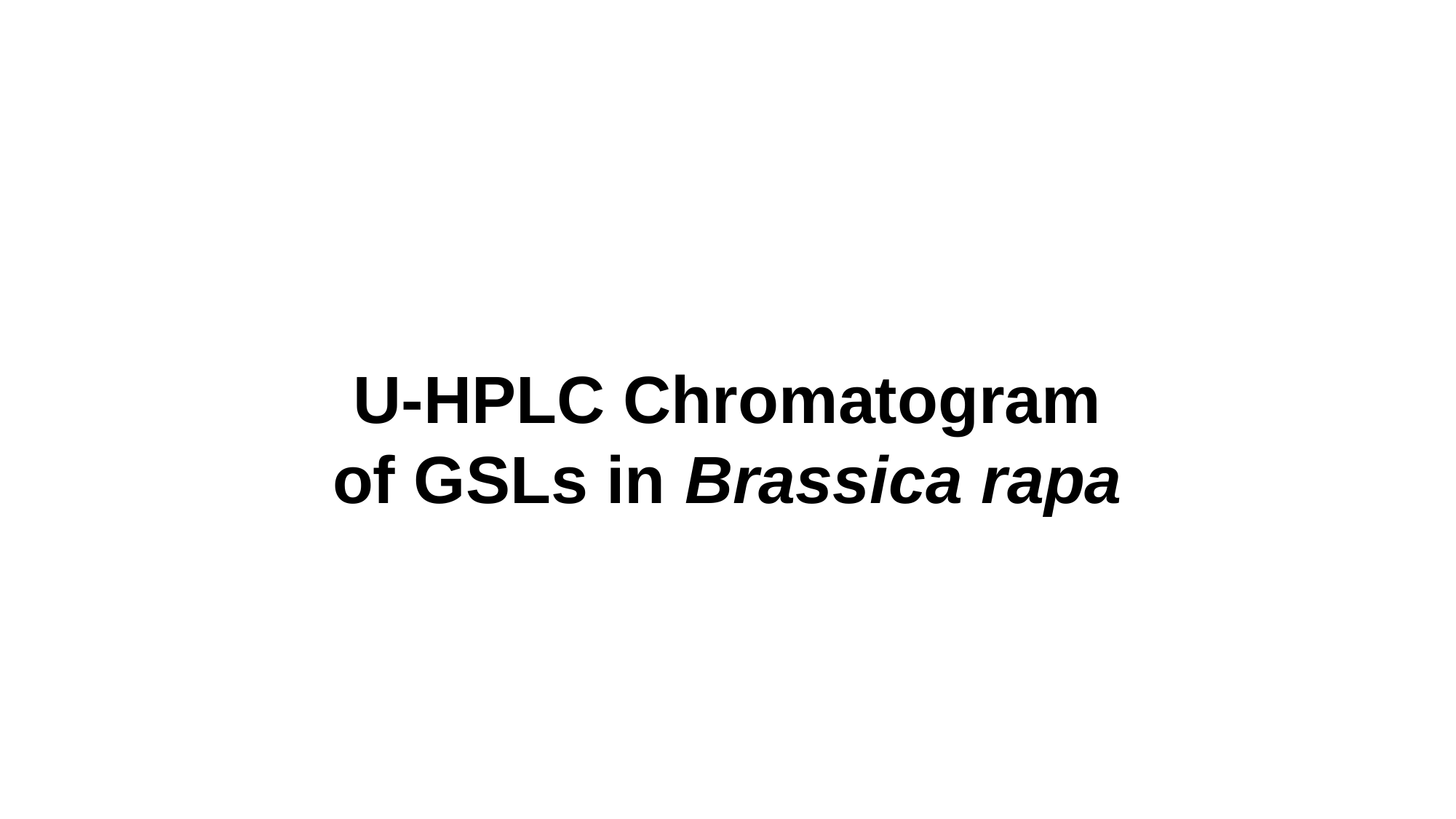

# U-HPLC Chromatogram of GSLs in Brassica rapa

## Slide 2
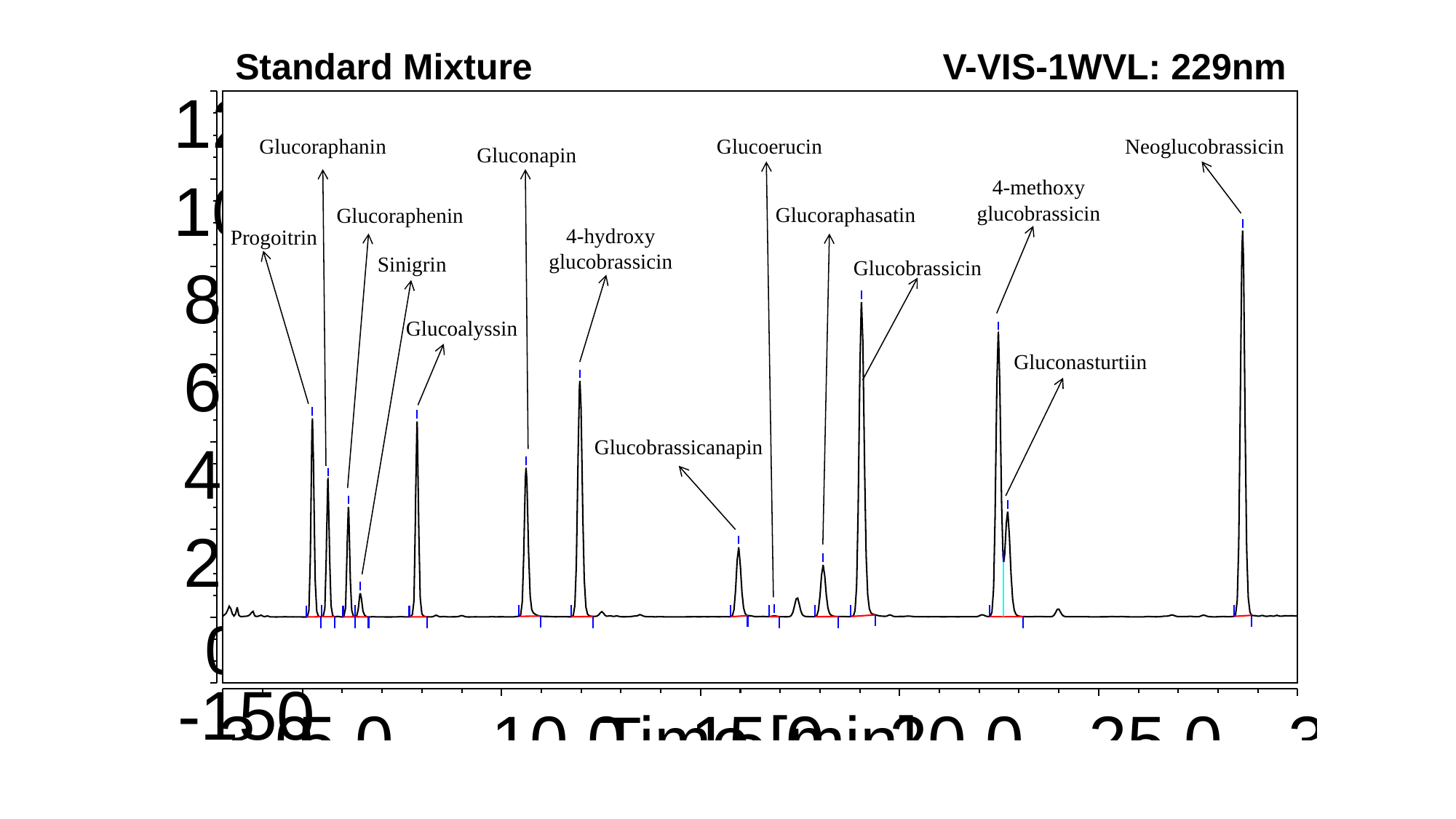

Standard Mixture			 	 V-VIS-1WVL: 229nm
Glucoraphanin
Neoglucobrassicin
Glucoerucin
Gluconapin
4-methoxy
glucobrassicin
Glucoraphenin
Glucoraphasatin
Progoitrin
4-hydroxy
glucobrassicin
Sinigrin
Glucobrassicin
Glucoalyssin
Gluconasturtiin
Glucobrassicanapin

## Slide 3
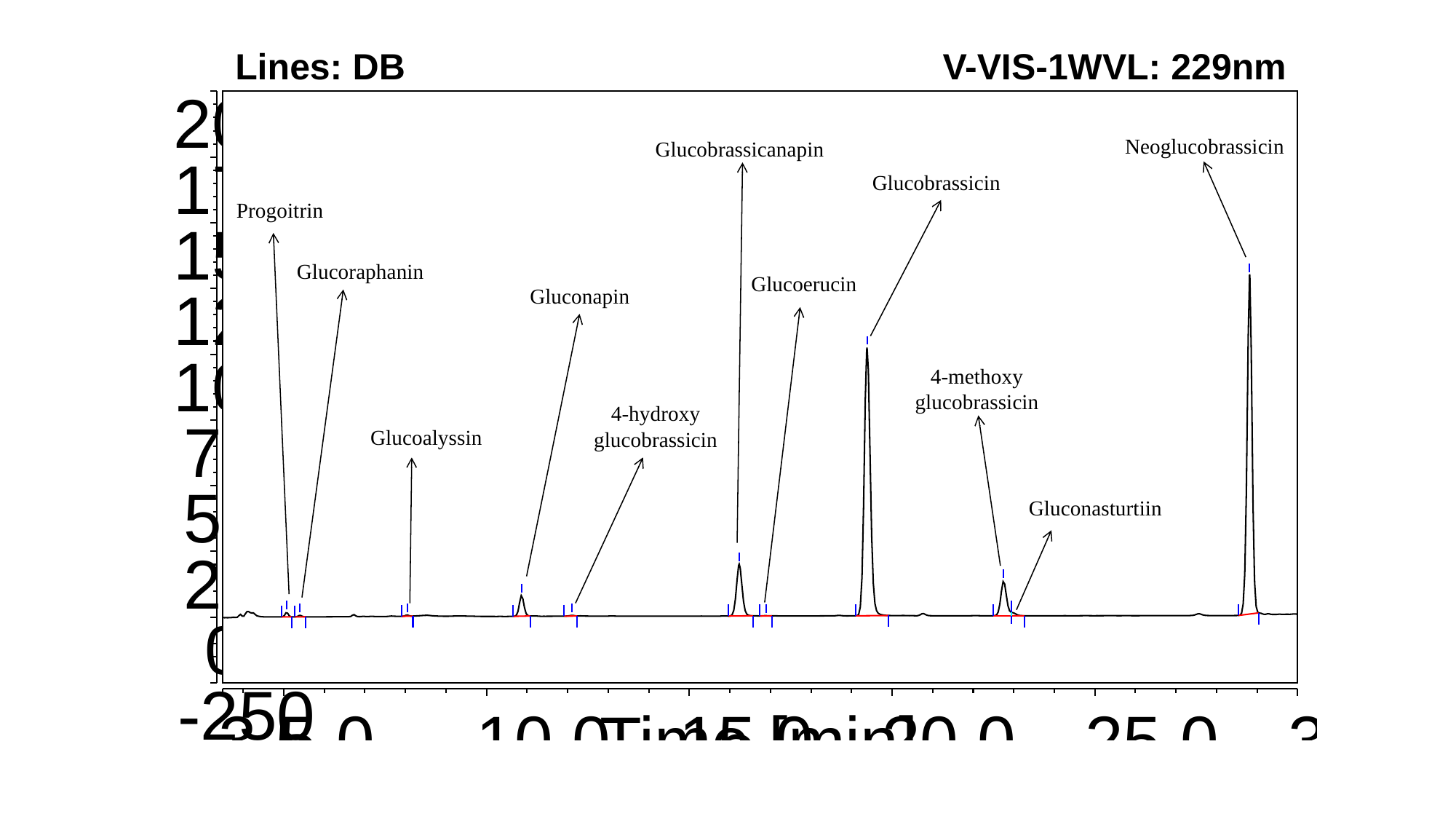

Lines: DB				 	 V-VIS-1WVL: 229nm
Neoglucobrassicin
Glucobrassicanapin
Glucobrassicin
Progoitrin
Glucoraphanin
Glucoerucin
Gluconapin
4-methoxy
glucobrassicin
4-hydroxy
glucobrassicin
Glucoalyssin
Gluconasturtiin

## Slide 4
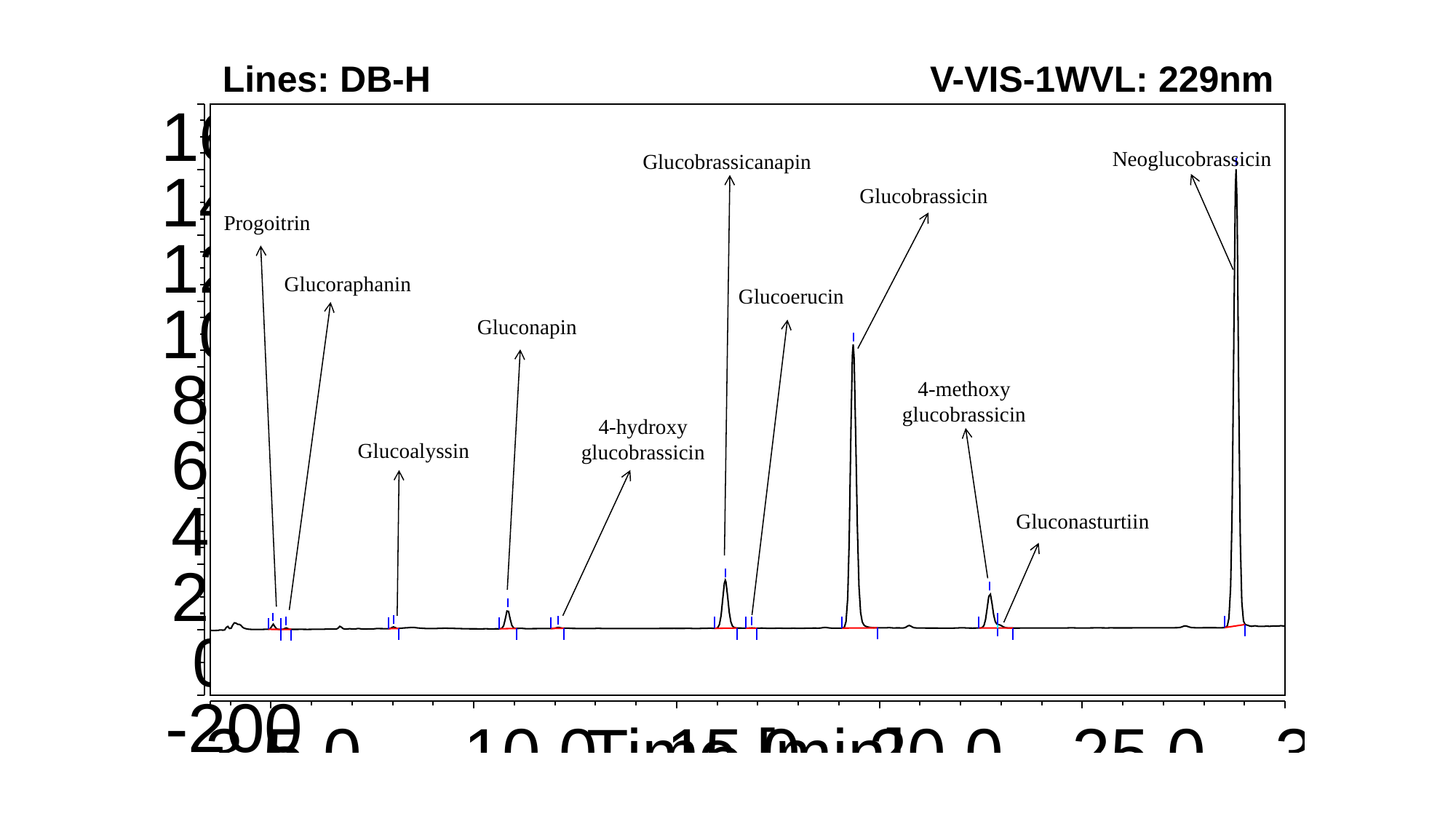

Lines: DB-H				 	 V-VIS-1WVL: 229nm
Neoglucobrassicin
Glucobrassicanapin
Glucobrassicin
Progoitrin
Glucoraphanin
Glucoerucin
Gluconapin
4-methoxy
glucobrassicin
4-hydroxy
glucobrassicin
Glucoalyssin
Gluconasturtiin

## Slide 5
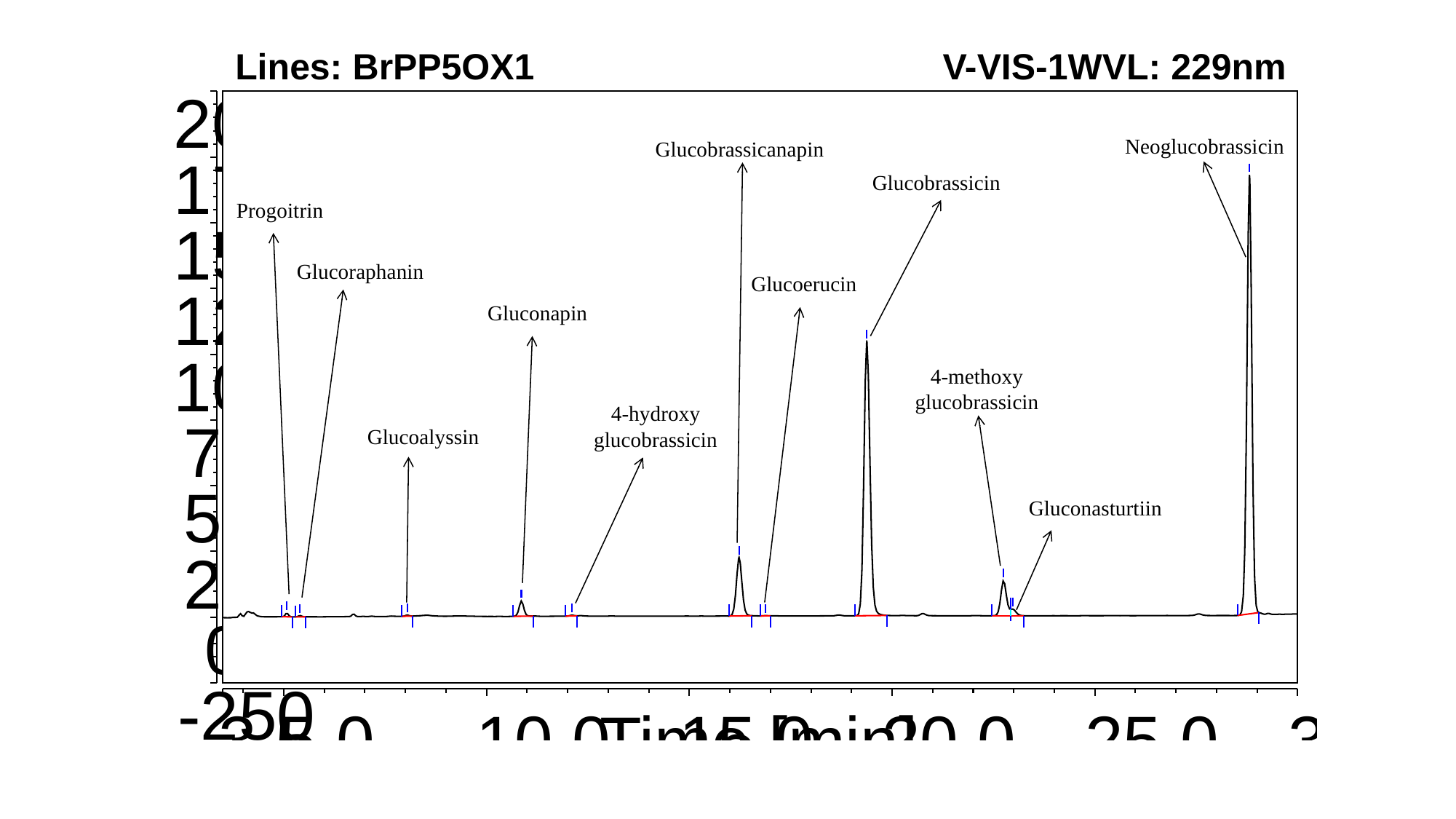

Lines: BrPP5OX1 		 	 V-VIS-1WVL: 229nm
Neoglucobrassicin
Glucobrassicanapin
Glucobrassicin
Progoitrin
Glucoraphanin
Glucoerucin
Gluconapin
4-methoxy
glucobrassicin
4-hydroxy
glucobrassicin
Glucoalyssin
Gluconasturtiin

## Slide 6
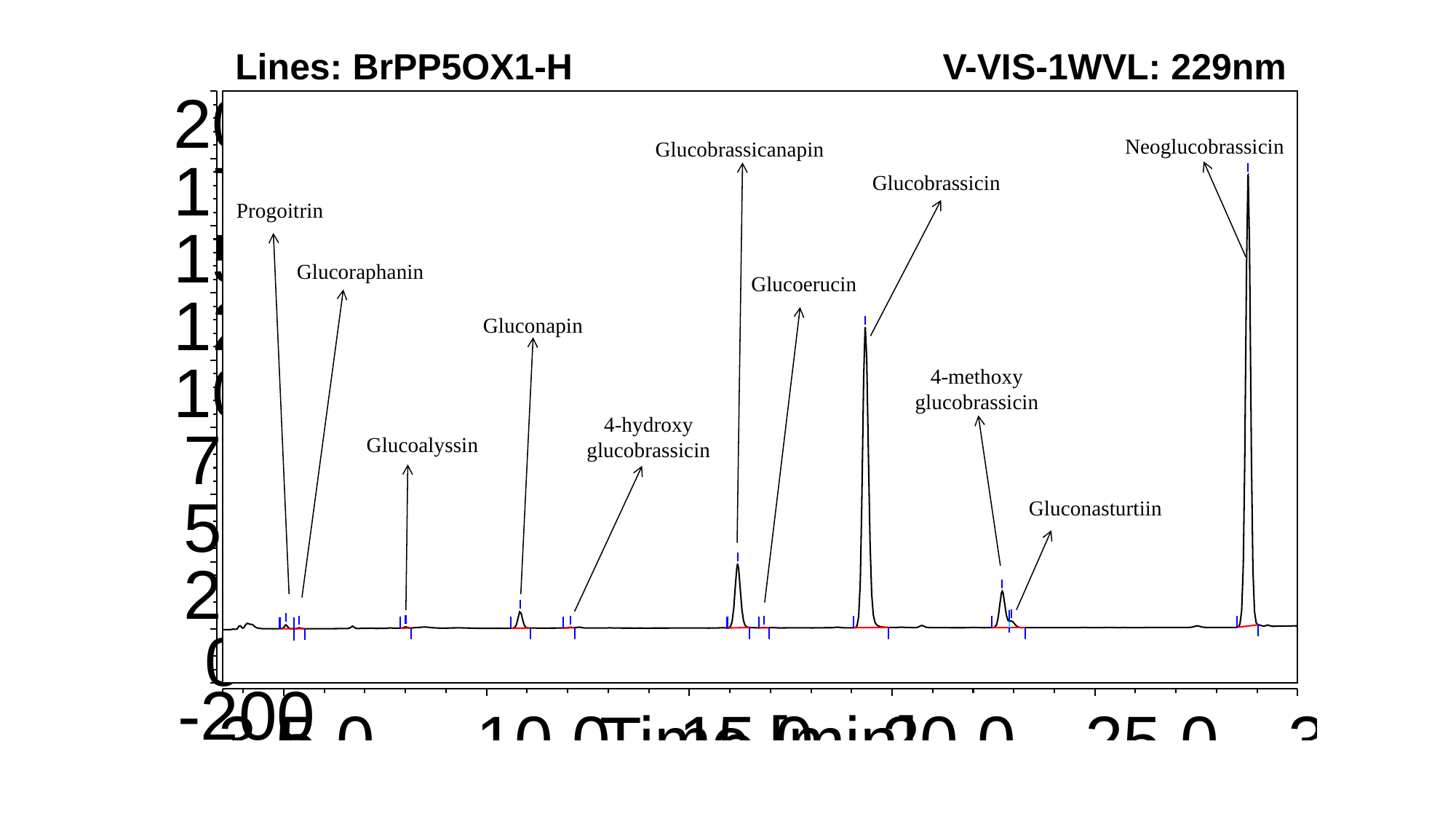

Lines: BrPP5OX1-H		 	 V-VIS-1WVL: 229nm
Neoglucobrassicin
Glucobrassicanapin
Glucobrassicin
Progoitrin
Glucoraphanin
Glucoerucin
Gluconapin
4-methoxy
glucobrassicin
4-hydroxy
glucobrassicin
Glucoalyssin
Gluconasturtiin

## Slide 7
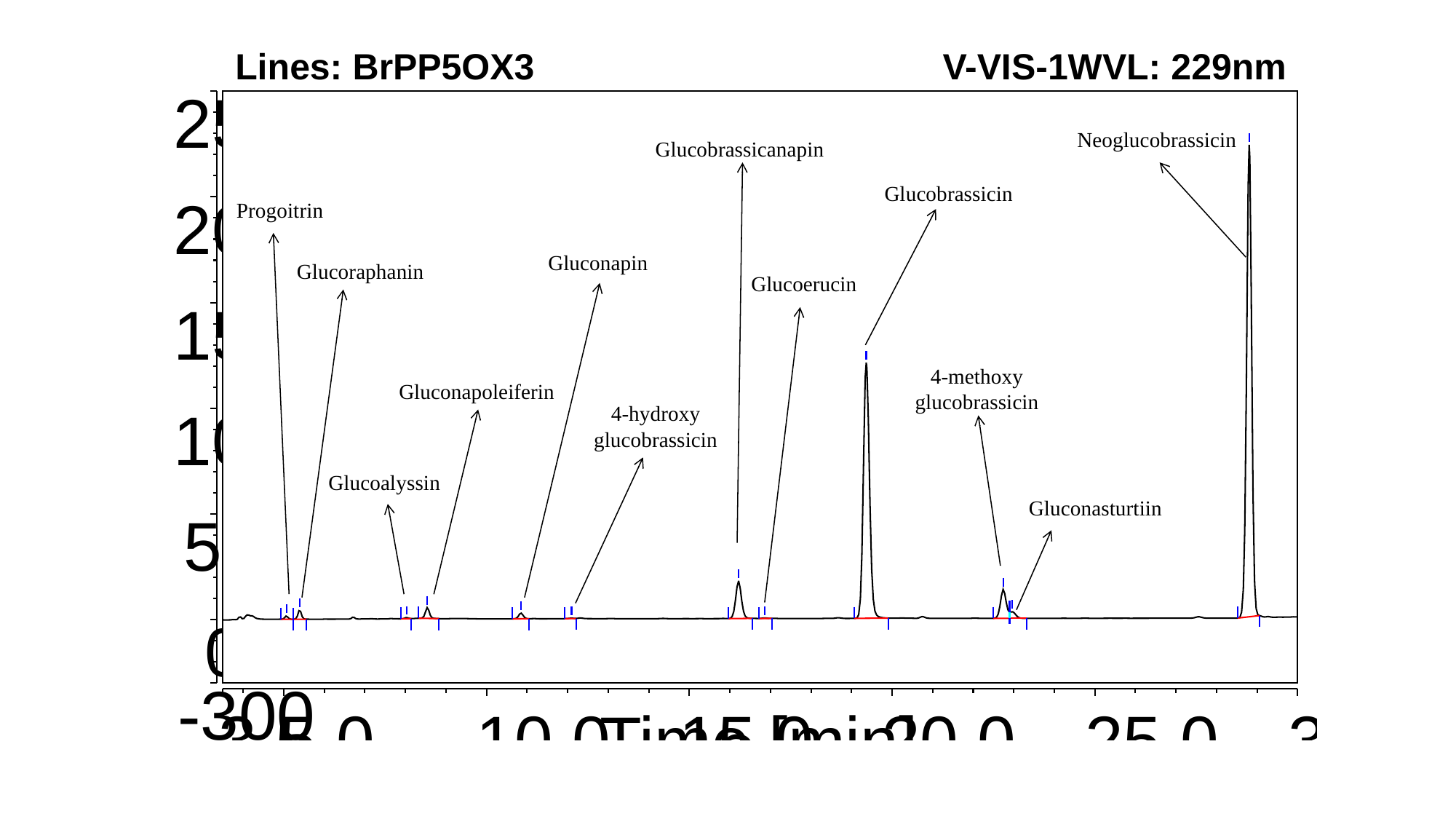

Lines: BrPP5OX3			 	 V-VIS-1WVL: 229nm
Neoglucobrassicin
Glucobrassicanapin
Glucobrassicin
Progoitrin
Gluconapin
Glucoraphanin
Glucoerucin
4-methoxy
glucobrassicin
Gluconapoleiferin
4-hydroxy
glucobrassicin
Glucoalyssin
Gluconasturtiin

## Slide 8
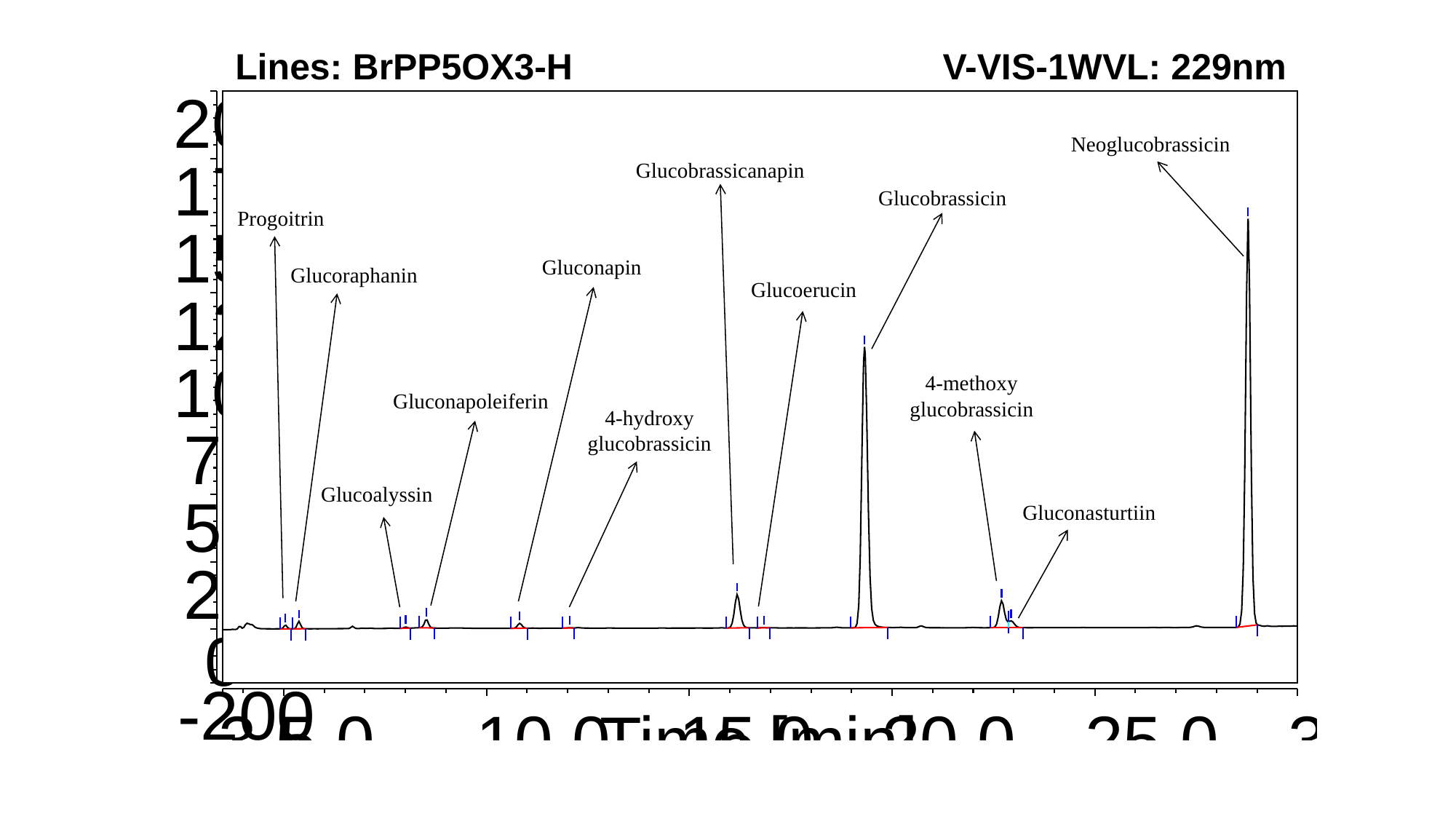

Lines: BrPP5OX3-H		 	 V-VIS-1WVL: 229nm
Neoglucobrassicin
Glucobrassicanapin
Glucobrassicin
Progoitrin
Gluconapin
Glucoraphanin
Glucoerucin
4-methoxy
glucobrassicin
Gluconapoleiferin
4-hydroxy
glucobrassicin
Glucoalyssin
Gluconasturtiin
