# Supplementary figures and images for: BrPP5.2 Overexpression Confers Heat Shock Tolerance in Transgenic Brassica rapa through Inherent Chaperone Activity, Induced Glucosinolate Biosynthesis, and Differential Regulation of Abiotic Stress Response Genes
Source: Int J Mol Sci. 2021 Jun 16;22(12):6437. doi: 10.3390/ijms22126437 (PMC8234546; doi:10.3390/ijms22126437)

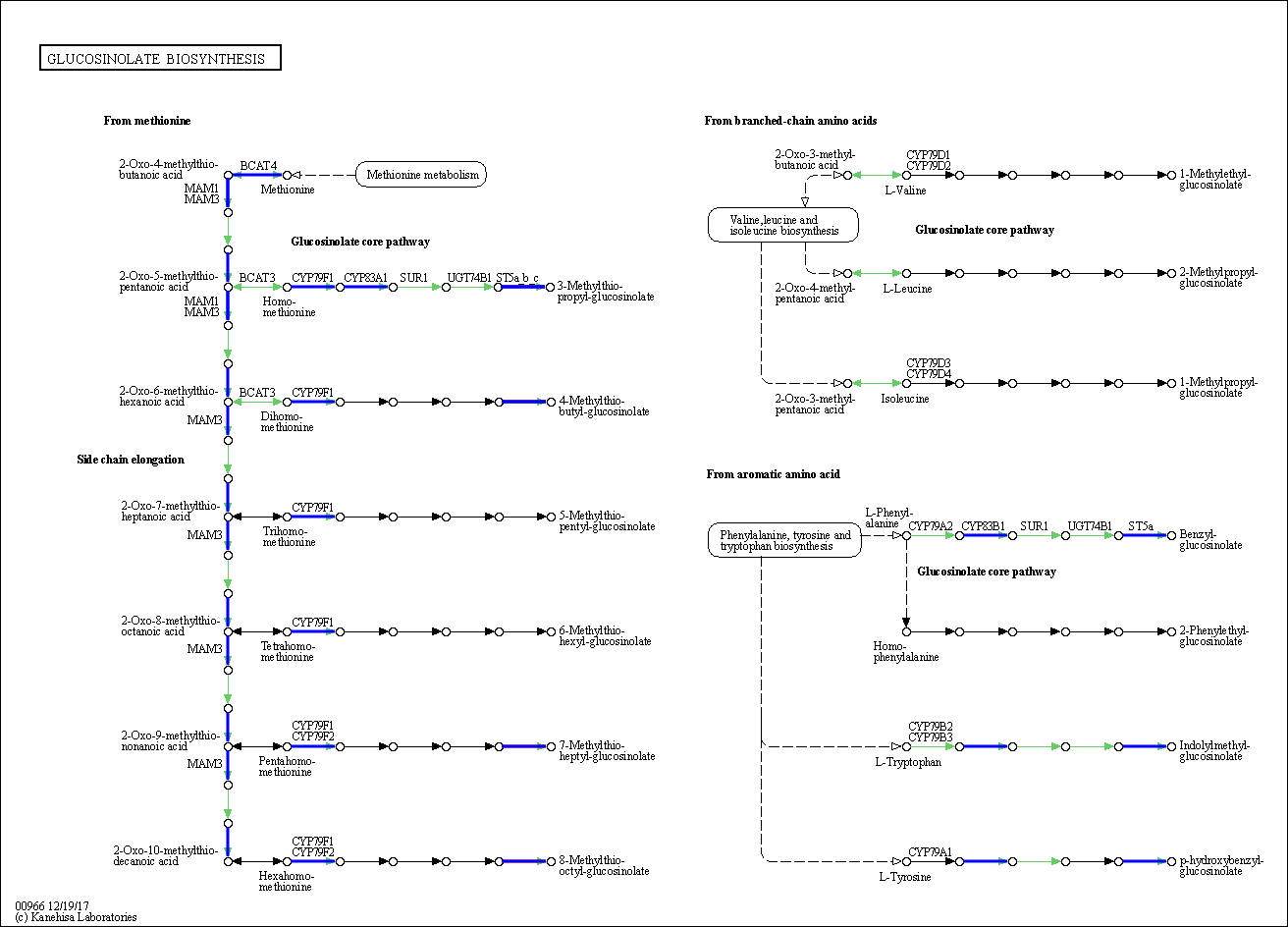

Supplement: Supplementary file 1 [file ijms-22-06437-s001.zip › ath00966 (1).png]
